# Co-encapsulation of hepatocytes, mesenchymal stem cells and growth factor in arginine-glycine-aspartate functionalized microbeads for liver disease

**DOI:** 10.1093/rb/rbaf094

**Published:** 2025-09-16

**Authors:** Su Yee Win, Pinunta Nittayacharn, Arkhom Saingam, Khanit Sa-ngiamsuntorn, Norased Nasongkla

**Affiliations:** Department of Biomedical Engineering, Faculty of Engineering, Mahidol University, Nakhon Pathom 73170, Thailand; Department of Biomedical Engineering, Faculty of Engineering, Mahidol University, Nakhon Pathom 73170, Thailand; Cryoviva (Thailand) Co., Ltd, Khunkaew Nakhonchaisri, Nakhon Pathom 73120, Thailand; Department of Biochemistry, Faculty of Pharmacy, Mahidol University, Bangkok 10400, Thailand; Department of Biomedical Engineering, Faculty of Engineering, Mahidol University, Nakhon Pathom 73170, Thailand

**Keywords:** cell encapsulation, basic fibroblast growth factor, stem cell, immortalized hepatocyte, click-RGD microbeads

## Abstract

Acute liver failure is a life-threatening condition with limited treatment options, primarily liver transplantation, which is constrained by donor shortages and lifelong immunosuppression. This study presents a minimally invasive therapeutic approach using multifunctional microbeads co-encapsulating two cell types: immortalized hepatocytes and umbilical cord-derived mesenchymal stem cells, along with basic fibroblast growth factor-loaded poly(lactide-co-glycolide) microspheres. The alginate microbeads are functionalized with poly(ethylene glycol) and the arginine-glycine-aspartate tripeptide to enhance cell adhesion and are crosslinked via click chemistry for improved structural integrity. The bFGF-loaded PLGA microspheres were synthesized using a double-emulsion solvent evaporation method, achieving an average size of 4.25 ± 2.20 µm, a loading content of 0.078% and an entrapment efficiency of 3.52 ± 0.27%. Sustained bFGF release over 14 days (cumulative 2.39 ± 0.20 ng) enhanced hepatocyte proliferation, human mesenchymal stem cell differentiation and cell viability. Functional assessment demonstrated significantly improved hepatocyte performance, with microbeads producing 2032.53 ± 29.45 ng of albumin and 1057.00 ± 9.19 ng of alpha-fetoprotein over 14 days. Overall, this co-encapsulation strategy enhances hepatocyte regeneration, viability, function and offers a scalable therapeutic platform for ALF. Future studies should optimize the formulation and evaluate long-term efficacy *in vivo*.

## Graphical abstract

**Figure:**
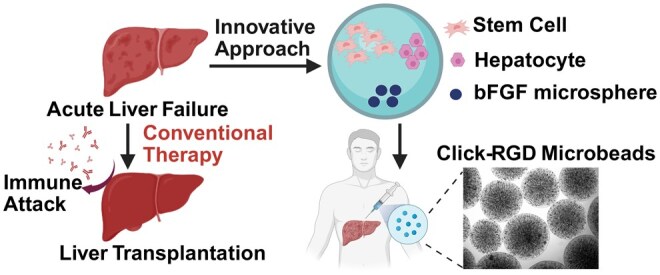

## Introduction

Acute liver failure (ALF) is a life-threatening condition characterized by the rapid loss of liver function, leading to coagulopathy, encephalopathy and multi-organ failure [1]. ALF can result from viral infections, drug-induced liver injury, autoimmune hepatitis or metabolic diseases. The rapid progression of ALF often results in patients with limited treatment options, and without timely intervention, mortality rates remain high [2, 3]. Liver transplantation remains the only curative option, significantly improving survival rates [4, 5]. However, limited donor availability, surgical risks and the need for lifelong immunosuppression restrict its accessibility. Alternative strategies like split liver transplants and living donor transplants have been explored but remain insufficient to meet demand [4]. Consequently, cell-based therapy has emerged as a promising alternative, offering a regenerative approach to restoring hepatic function through the engineering of artificial organs.

Among various cell-based innovations, cell encapsulation has emerged as a promising alternative therapy for ALF, providing temporary hepatic function while protecting encapsulated cells from immune rejection [6]. This approach involves encapsulating hepatocytes within a biocompatible, semi-permeable hydrogel matrix, allowing the controlled exchange of nutrients, oxygen and metabolic waste while shielding cells from the host immune system. Encapsulated hepatocytes retain their ability to synthesize essential liver-specific proteins, such as albumin and alpha fetoprotein (AFP), and perform metabolic functions without requiring immunosuppression [7]. Unlike traditional hepatocyte transplantation, encapsulation enhances cell survival, proliferation and functionality by providing a controlled microenvironment [2, 4].

Among cell encapsulation strategies, alginate microbeads have been widely explored due to their biocompatibility, ease of gelation and tunable porosity. They have successfully encapsulated primary hepatocytes and human mesenchymal stem cells (hMSCs) to restore liver function. hMSCs offer additional therapeutic benefits, including immunomodulation, anti-inflammatory properties and differentiation into hepatocyte-like cells [8, 9]. Despite these advantages, conventional alginate microbeads have critical limitations that hinder their clinical application. Alginate beads lack structural integrity, resulting in rapid degradation and loss of cell protection, which exposes encapsulated cells to immune attack. Poor cell adhesion within alginate matrices limits hepatocyte viability and function [10].

Additionally, hepatocyte encapsulation alone may not be sufficient for long-term liver regeneration, as hepatocyte proliferation and differentiation require biochemical cues, such as growth factors. Addressing these challenges requires a multifunctional encapsulation system that enhances mechanical integrity, cell adhesion and controlled release of growth factors to sustain hepatocyte survival and function. Our previous work developed a co-encapsulation system of basic fibroblast growth factor (bFGF)-loaded poly(lactide-co-glycolide)(PLGA) microspheres with hepatocytes, demonstrating that sustained growth factor delivery can effectively prolong hepatocyte viability and function [11]. We also expanded the application of alginate microbeads by encapsulating CsA-loaded PLGA nanospheres, thereby achieving controlled drug release and local immunosuppression [12]. Together, these studies highlighted the potential of alginate-based microbeads to support cell viability, structural integrity and controlled therapeutic delivery when properly engineered.

Building upon our previous work, this study proposes a novel encapsulation system that integrates click-arginine-glycine-aspartate tripeptide (RGD) microbeads, bFGF-loaded PLGA microspheres, immortalized hepatocytes (imHCs) and hMSCs. In this study, click-RGD microbeads are synthesized via Copper(I)-catalyzed azide-alkyne cycloaddition (CuAAC) reaction, incorporating RGD peptides into a poly(ethylene glycol) (PEG)-crosslinked alginate matrix [13]. bFGF is crucial for hepatocyte proliferation and the differentiation of hMSCs into hepatocyte-like cells. Its encapsulation in PLGA microspheres enables controlled and sustained release, ensuring a continuous supply of growth factors to promote long-term cell viability and liver regeneration [14, 15]. Unlike primary hepatocytes, novel imHCs were derived from hMSCs. It provides a stable, reproducible, functional hepatocyte source with prolonged viability and sustained albumin and AFP production. Additionally, hMSCs contribute additional immunomodulatory, paracrine and regenerative effects, further supporting hepatocyte function and survival [16, 17].

**Figure 1.**
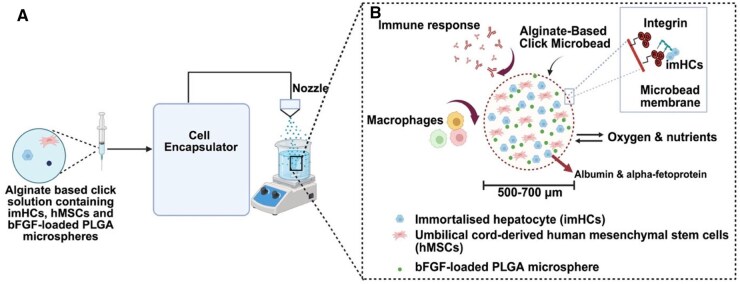
Schematic diagram illustrating the concept of RGD-functionalized click microbeads for growth factor and cell co-encapsulation. (**A**) Microbeads are fabricated via electrostatic extrusion. (**B**) The resulting homogeneous microbeads (500–700 µm) encapsulate two cell types (imHCs and hMSCs) and bFGF-loaded PLGA microspheres. The semipermeable membrane enables oxygen and nutrient exchange, supporting the functionality of imHCs and cytokine-mediated hepatocyte regeneration. Created with Biorender.com.

We hypothesize that integrating these bioengineering strategies into a single encapsulation system will synergistically improve hepatocyte viability, proliferation and liver-specific function, offering a scalable and minimally invasive alternative to liver transplantation. A schematic representation of the encapsulation system is provided in Figure 1. To test this hypothesis, this study investigates (1) the synthesis and characterization of bFGF-loaded PLGA microspheres for sustained growth factor release, (2) the fabrication of click-RGD microbeads with enhanced structural integrity, (3) the evaluation of encapsulated hepatocyte and hMSCs viability, proliferation and differentiation and (4) the assessment of hepatocyte-specific function, including albumin and AFP production. This study aims to establish a clinically translatable encapsulation system for ALF treatment by integrating biochemical, mechanical and regenerative enhancements.

## Materials and methods

### Materials

Sodium alginate was purchased from BUCHI, Switzerland and calcium chloride was obtained from Ajax Finechem Pty Ltd, Australia. HEPES, dithiothreitol (DTT) and sucrose were sourced from Sigma-Aldrich, USA, while Siam Bioscience, Thailand, supplied bFGF. PLGA (75:25, MW 32 kDa) and PEG were obtained from NanoPolyPEG, Co., Ltd, Thailand. Poly(vinyl alcohol) (PVA) was purchased from Sigma-Aldrich, Germany. Dichloromethane (DCM) was obtained from Honeywell Burdick & Jackson, Korea. Tris(hydroxymethyl)aminomethane hydrochloride (Tris-HCl) was sourced from EMD Millipore, Germany, while sodium chloride was purchased from EMD Millipore, USA. Ethylenediaminetetraacetic acid disodium salt dihydrate (EDTA) was obtained from Amresco, USA. Cell culture reagents, including Dulbecco’s Modified Eagle’s Medium/Nutrient Mixture F12 (DMEM/F12), penicillin-streptomycin, trypsin, Dulbecco’s Modified Eagle’s Medium (DMEM, low glucose) and TrypLE™ Express Enzyme, were all obtained from Gibco, USA. Fetal bovine serum (FBS) was purchased from Gibco, UK. Carboxyfluorescein diacetate succinimidyl ester (CFSE, ab113853) was sourced from Abcam, UK. Hoechst 33342 and PI/RNase staining solution (FxCycle) were purchased from Thermo Fisher Scientific, USA. Human Albumin ELISA Kit (Catalog No. MBS564029) and Human AFP ELISA Kit (Catalog No. MBS165016) were obtained from MyBioSource, USA. Tosyl chloride, sodium azide and sodium ascorbate were purchased from Tokyo Chemical Industry, Japan. Copper (II) sulfate pentahydrate was obtained from ACROS, Belgium. GGGGRGDSP peptide was purchased from Biomatik, USA. Sodium hydroxide (NaOH), tetrahydrofuran, diethyl ether, dichloromethane, dimethylformamide, N-hydroxysuccinimide (NHS), 1-ethyl-3-(3-dimethylaminopropyl) carbodiimide (EDC) and propargyl amine (PA) were sourced from Sigma-Aldrich, USA. hMSCs were provided by Cryoviva (Thailand) Co., Ltd (Ethical approval: MU-CIRB 2023/236.2707). imHCs (ATCC, Manassas, VA, USA) were obtained from Assistant Professor Khanit Sa-ngiamsuntorn, Mahidol University (Ethical approval: MU-CIRB 2023/236.2707).

### Preparation of bFGF-loaded PLGA microspheres

The methodology used in this study was primarily adapted from previously published work [11]. Blank (unloaded) and bFGF-loaded PLGA microspheres were prepared using the double emulsion solvent evaporation method (W_1_/O/W_2_) as described previously [18–20]. The preparation process was identical for both formulations, except that the blank microspheres were prepared without bFGF in the aqueous phase. For bFGF-loaded PLGA microspheres, a bFGF solution (10 µg/mL) was prepared in 1 mL of buffer containing 20 mM Tris-HCl, 150 mM NaCl, 3 mM DTT, 1 mM EDTA and 2% (w/v) sucrose. This aqueous phase (W_1_) was added to PLGA dissolved in DCM (O phase) and homogenized at 12 000 rpm for 2 min to form the primary emulsion (W_1_/O). The W_1_/O emulsion was then emulsified in 20 mL of 2% (w/v) PVA (W_2_ phase) and homogenized at 18 000 rpm for 5 min, creating the W_1_/O/W_2_ double emulsion. The emulsion was stirred overnight to evaporate DCM. Microspheres were purified by centrifugation at 14 000 rpm for 40 min, followed by three washes with deionized (DI) water to remove free bFGF and residual reagents. The purified microspheres were frozen at −20°C and lyophilized, yielding a dry microsphere powder for further use and characterization.

### Characterization of PLGA microspheres

#### Physical properties

The size distribution of blank and bFGF-loaded PLGA microspheres was analyzed using dynamic light scattering (DLS) with a particle size analyzer (Partica Mini LA-350, HORIBA) as described in this article [21, 22]. To prepare the samples, microsphere powders were resuspended in DI water and filtered through 11-µm Whatman filter papers to remove large polymer residues. The filtered suspension was then diluted at a 1:10 ratio, and size measurements were performed in triplicate (*n* = 3) for each sample. Three consecutive runs were conducted for each measurement, and the average particle size was calculated and recorded. Additionally, the size, shape and surface morphology of the microspheres were further examined using scanning electron microscopy (SEM) (JSM-IT500HR InTouchScope™, JEOL, Ltd) [19, 23]. For SEM analysis, freeze-dried microsphere samples were mounted onto a sample holder and coated with a thin layer of gold to enhance conductivity. The coated samples were then imaged under a high vacuum at an accelerating voltage of 20 kV to assess surface characteristics and structural integrity at 4000× and 12 000× magnifications.

#### Production yield, loading content and encapsulation efficiency

The loading efficiency of bFGF in PLGA microspheres was quantified using a phase separation method. Briefly, 10 mg of bFGF-loaded PLGA microspheres were dissolved in 1 mL of dichloromethane in a separation funnel. To induce phase separation, 3 mL of DI water was added and the mixture was vigorously shaken multiple times to ensure thorough extraction. The solution was then left undisturbed for 3 h to allow complete separation of the aqueous and organic phases. Due to its hydrophilic nature, bFGF preferentially partitions into the aqueous phase. The aqueous phase was carefully collected, and bFGF concentration was quantified using a bFGF ELISA Kit according to the manufacturer’s instructions. The yield, loading content and encapsulation efficiency were calculated using Equations (1)–(3).


(1) $$\mathrm{Yield}\;(\%)\;=\frac{\mathrm{Weight}\;\mathrm{of}\;\mathrm{microspheres}}{\mathrm{Total}\;\mathrm{weight}\;\mathrm{of}\;\mathrm{initial}\;\mathrm{polymer}\;\mathrm{and}\;\mathrm{bFGF}}\;\times \;100\%$$

(2) $$\mathrm{Loading}\;\mathrm{Content}\;(\%)\;=\;\frac{\mathrm{Weight}\;\mathrm{of}\;\mathrm{bFGF}\;\mathrm{in}\;\mathrm{microspheres}\;}{\mathrm{Weight}\;\mathrm{of}\;\mathrm{microspheres}}\;\times \;100\%$$

(3) $$\mathrm{Encapsulation}\;\mathrm{Efficiency}\;(\%)=\;\frac{\mathrm{Weight}\;\mathrm{of}\;\mathrm{bFGF}\;\mathrm{in}\;\mathrm{microspheres}}{\mathrm{Weight}\;\mathrm{of}\;\mathrm{initial}\;\mathrm{bFGF}}\;\times \;100\%$$

#### bFGF release kinetics from microspheres

The release of bFGF from microspheres was evaluated in triplicate using a dialysis membrane. Microspheres (38.5 mg) were suspended in 1 mL of DI water and incubated in a shaker at 37°C to simulate physiological conditions at predetermined time points (1, 6, 12 and 24 h; 3, 7, 10 and 14 days), 0.5 mL of samples were collected and immediately replaced with an equal volume of fresh DI water to maintain constant volume conditions. The collected samples were centrifuged at 18 000 rpm for 45 min to separate the released bFGF in the supernatant. The bFGF concentration in the supernatant was quantified using enzyme-linked immunosorbent assay (ELISA), with O.D. measurements at 450 nm. The bFGF concentration was determined by interpolating the O.D. values from a standard calibration curve.

### Click microbead formulation

To achieve a click crosslinking system, three key components are synthesized: PEG-Azide, which provides the azide (-N_3_) functionality necessary for CuAAC, and alginate-alkyne and alginate-RGD, which introduce the required alkyne (-C≡CH) groups for efficient crosslinking. The synthesis of these functionalized polymers is described in the following sections.

#### PEG-azide synthesis

PEG-azide was synthesized from hydroxyl-terminated PEG via a PEG-tosyl intermediate, which facilitates the conversion of hydroxyl groups to azide. To synthesize PEG-tosyl, hydroxyl-terminated PEG was dissolved in DCM and stirred overnight. Triethylamine was then added, and the mixture was stirred for 36 h at an ice bath temperature. The solvent was removed using a rotary evaporator, and the resulting powder was dissolved in 100 mL of DI water for purification. The aqueous phase was extracted with diethyl ether, followed by DCM extraction. The DCM phase was dried over MgSO_4_, and the product was precipitated with diethyl ether and then evaporated. For azide modification, PEG-tosyl was dissolved in ethanol, followed by the addition of sodium azide (NaN_3_). The reaction mixture was refluxed for 16 h. Ethanol was removed using a rotary evaporator, and 50 mL of acetone was added to eliminate unreacted azide. The mixture was then centrifuged at 3000 rpm for 10 min to further purify the product.

#### Alginate-alkyne and alginate-RGD synthesis

Alginate-alkyne and alginate-RGD were synthesized by activating the carboxyl groups of alginates using N-hydroxysuccinimide (NHS) and 1-ethyl-3-(3-dimethylaminopropyl) carbodiimide (EDC), enabling conjugation with alkyne and RGD groups, respectively [24]. For both modifications, the alginate solution (1% w/v) was mixed with NHS and EDC and then stirred for 30 min at room temperature. For the alginate-alkyne reaction, propargylamine (PA) was added and the reaction was continued overnight. For alginate-RGD, the RGD peptide was added, and the reaction was stirred overnight. Each reaction mixture was then dialyzed in DI water using a 50 kDa molecular weight cut-off (MWCO) dialysis membrane for four days, followed by freeze-drying to obtain the final product.

#### ^1^H NMR characterization

The chemical compositions of the click reaction, including PEG-azide, alginate-alkyne and alginate-RGD, were analyzed using Proton Nuclear Magnetic Resonance spectroscopy (^1^H NMR) (BRUKER brand, Model: 600 MHz + Cyro probe). To ensure complete solubilization and optimal spectral resolution for ^1^H NMR analysis, 15 mg of PEG-azide was dissolved in 600 µl of chloroform-D, whereas 15 mg of alginate-alkyne and alginate-RGD were each dissolved in 600 µl of deuterium oxide (D_2_O). A total of 512 scans were accumulated for each sample to obtain high-resolution spectra suitable for structural elucidation. The chemical shifts and peak integrations were analyzed to confirm the molecular structures and the presence of functional groups relevant to the click reaction. This ^1^H NMR analysis provided essential verification of the chemical identity and purity of the reactants, ensuring the reliability of subsequent click-RGD microbead synthesis.

### Cell culture

imHCs were cultured in DMEM/F12 medium supplemented with 10% FBS and 1% penicillin-streptomycin. hMSCs were maintained in a DMEM/low glucose medium with the same supplementation. Both cell types were incubated at 37°C in a humidified atmosphere containing 5% CO_2_ [25]. Culture media were replaced every three days to maintain optimal growth conditions for the cells. Cells were passaged at 80–90% confluency using 0.125% trypsin-EDTA for detachment. The detached cells were centrifuged at 1500 rpm for 6 min at 4°C, washed with fresh medium to remove residual trypsin, and resuspended for subsequent use. The concentration of cells was determined using the trypan blue exclusion assay to ensure cell viability and accurate cell counts.

### Fabrication of alginate-based click microbeads

**Figure 2.**
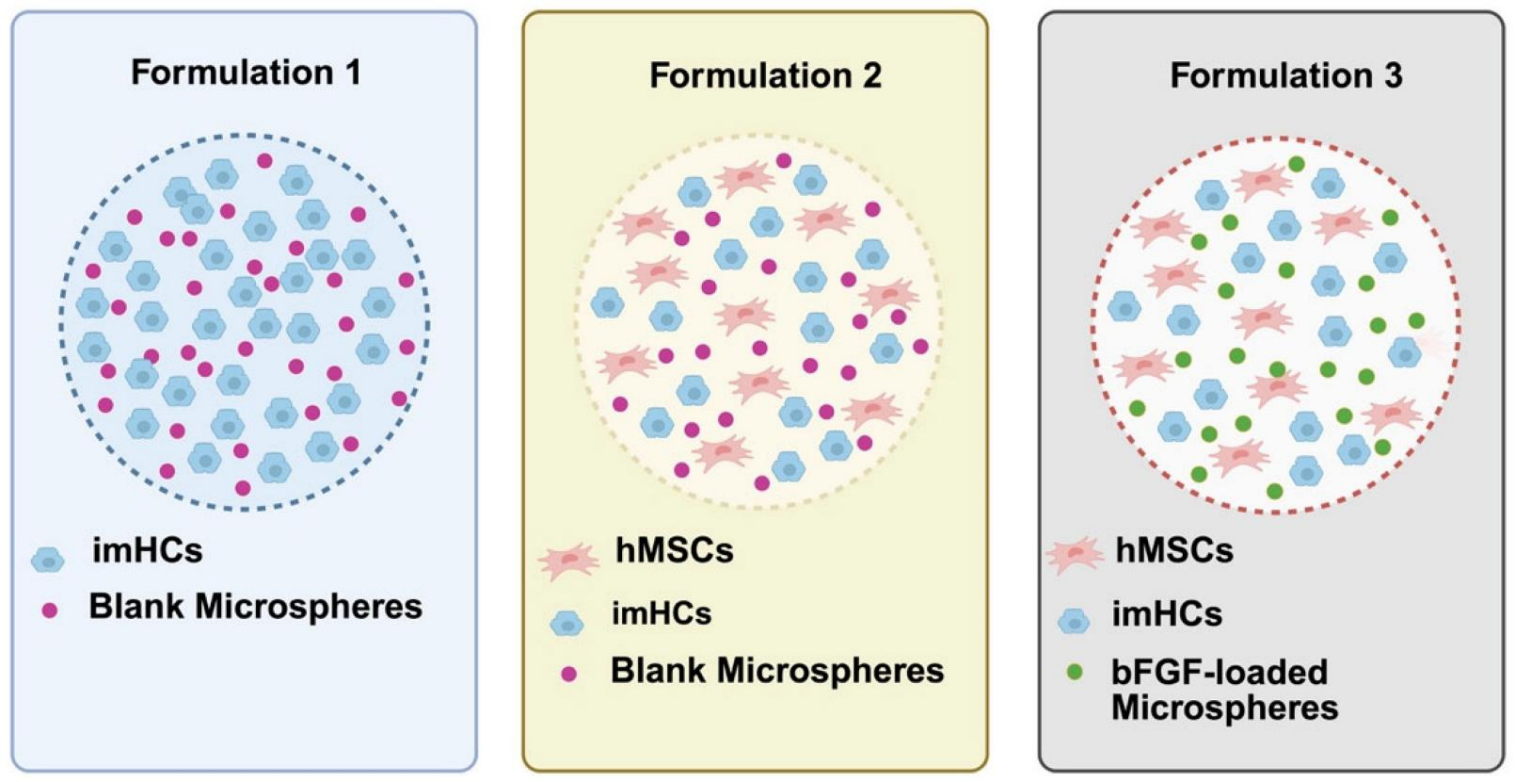
Schematic representation of the alginate-based click microbead formulations used in this study. Formulation 1 (F1) consists of microbeads encapsulating imHCs and blank PLGA microspheres. Formulation 2 (F2) includes microbeads encapsulating hMSCs, imHCs and blank PLGA microspheres. Formulation 3 (F3) contains microbeads encapsulating hMSCs, imHCs and bFGF-loaded PLGA microspheres. The alginate concentration, cell number and microsphere density remain consistent across all formulations. Created with Biorender.com.

Alginate-based click microbeads modified with PEG and RGD were fabricated using electrostatic extrusion for the encapsulation of two cell types and bFGF-loaded PLGA microspheres. Illustrations showing three different microbead formulations used in this study are shown in Figure 2. The detailed formulations used in this study are summarized in Table 1. To prepare 5 mL of the alginate solution, 1.5% w/v alginate was dissolved in a sodium chloride solution containing PEG-azide, alginate-RGD and alginate-alkyne. The solution was stirred until fully dissolved and then filtered through a 0.4 µm cellulose acetate syringe filter. For formulations containing microspheres and cells, bFGF-loaded PLGA microspheres (1.5 mg/mL), imHCs (1 × 10^6^ cells/mL) and hMSCs (0.5 × 10^6^ cells/mL) were resuspended in the prepared alginate solution. The mixture was then extruded through a 300 µm nozzle using an Encapsulator (B-395 Pro, BÜCHI, Ireland) directly into a 115 mM calcium chloride (CaCl_2_) solution for gelation [11]. Immediately after extrusion, 0.1 mM sodium-L-ascorbate and 0.01 mM copper (II) sulfate were gradually added dropwise into the gelling bath containing 115 mM CaCl_2_ and the extruded microbeads. The microbeads were stirred for an additional 15 min to facilitate Ca^2+^ and Na^+^ ion exchange and crosslinking, reinforcing the structural integrity of the click-alginate microbeads. Finally, the microbeads were collected, washed with DI water and cultured in DMEM/F12 for further use.

**Table 1.** Alginate-based click microbead formulations

Formulation	Click microbead composition (For 5 mL)	Cell line(s)	Type of microsphere
F1	Alginate 1.5 %w/v, 425 mg of PEG-azide (17 µM), 120 mg of alginate-RGD (110 µM) and 28 mg of alginate-alkyne (28 µM)	imHCs	Blank microsphere
F2		imHCs and MSCs	Blank microsphere
F3		imHCs and MSCs	bFGF-loaded microsphere

### Characterization of alginate-based click microbeads

#### Size and surface morphological analysis

The size and surface morphology of the fabricated microbeads were analyzed using an inverted light microscope and SE [26–28]. Microbeads were prepared using an encapsulator and imaged directly under an inverted microscope to assess bead formation, shape, size distribution and click reaction features. For SEM analysis, samples were dehydrated through a graded ethanol series (30, 50, 70, 90 and 100%), with each step lasting 10 min [29, 30]. This dehydration process was repeated three times to ensure complete removal of moisture. Following dehydration, the microbeads underwent critical point drying using a Leica EM CPD 300. Dried samples were mounted on grids and coated with a thin layer of gold using a Balzers SCD 040 sputter coater to enhance conductivity and imaging quality. SEM-EDS analysis was conducted using a JEOL JSM-IT500HR at an accelerating voltage of 20 kV.

#### Assessment of structural integrity of microbeads

The microbeads described in Table 1 were suspended in DMEM/F12 medium and incubated at 37°C with 5% CO_2_ in a humidified incubator. Their structural integrity was monitored over time using an inverted light microscope (Sky brand, Model: MT.02.DS.3), with size measurements recorded at Days 0, 1, 3, 7, 10 and 14 to assess the degradation and structural integrity of microbeads. The culture medium was replaced every three days to maintain consistent conditions. For each time point, images were captured from three different fields of view, and the size of 10 microbeads per image (*n* = 10) was measured using ImageJ software.

#### Cell distribution and viability

To assess cell viability and distribution within the microbeads, hMSCs were pre-stained with carboxyfluorescein diacetate succinimidyl ester (CFDA-SE), an amine-reactive, cell-permeable fluorescent dye, before encapsulation [31]. hMSCs (1 × 10^6^ cells) were incubated with 5 µM CFDA-SE in DMEM low-glucose medium at 37°C for 10–15 min in the dark, with gentle agitation every 5 min to ensure uniform staining. The reaction was quenched by adding an equal volume of complete medium containing 10% FBS, followed by an additional 5-min incubation. The labeled cells were centrifuged at 1200 rpm for 5 min. The supernatant was then discarded, and the resulting cell pellet was encapsulated in microbeads. The microbeads described in Table 1 were suspended in DMEM/F12 medium and incubated at 37°C with 5% CO_2_ in a humidified incubator. The culture medium was replaced every three days to maintain consistent conditions. At designated time points (Days 0, 1, 3, 7, 10 and 14), cells inside the microbead were subjected to Hoechst 33342 and propidium iodide (PI) staining to evaluate cell viability. Hoechst 33342 was applied at a final concentration of 6.5 µL/mL in DMEM/F12 and incubated at 37°C for 10 min, followed by PI staining (10 µL/mL in DMEM/F12) with an additional 10-min incubation at room temperature under light-protected conditions [32]. Excess dye was removed through two washes with DMEM/F12 to minimize background fluorescence. Following staining, fluorescence imaging was performed using a confocal microscope (LSM 800, Carl Zeiss, Jena, Germany) with z-stack confocal imaging (425 µm size, 48 slices, 9 µm interval). The laser power settings were 40% for CFSE, 50% for Hoechst 33342 and 20% for PI, with excitation/emission wavelengths set at 462 nm/517 nm for CFSE, 400 nm/461 nm for Hoechst and 518 nm/615 nm for PI. In this dual-staining approach, viable hMSCs exhibited green fluorescence (CFSE labeling), viable hepatocytes stained blue (Hoechst 33342) and non-viable cells displayed red fluorescence (PI). Finally, the number of viable cells within the microbeads was quantified at each time point using ImageJ software to assess cell viability and distribution.

#### Functionality of encapsulated imHCs in microbeads

To assess hepatocyte functionality, microbeads were incubated in a DMEM/low-glucose (DMEM/LG) medium supplemented with appropriate nutrients in a CO_2_ incubator. The culture supernatant was collected on Days 0, 1, 3, 7, 10 and 14 for analysis. The levels of albumin and AFP secreted by encapsulated imHCs were quantified using human albumin and AFP ELISA kits, providing a measure of hepatic function over time.

### Statistical analysis

All experiments were performed in triplicate, and data are presented as the mean ± standard error of the mean. Statistical comparisons between groups were conducted using a two-way analysis of variance (ANOVA) with repeated measures and multiple comparisons. *P* values ≤ 0.05 was considered statistically significant unless otherwise specified.

## Results

Click-RGD alginate microbeads co-encapsulating bFGF-loaded PLGA microspheres, imHCs and hMSCs were successfully developed in this study. To confirm the feasibility and effectiveness of the proposed encapsulation system, a series of experiments was conducted to evaluate its structural properties, controlled release behavior and biological performance. The evaluation included microsphere characterizations, microbead morphology, structural integrity assessment and analysis of cell viability and hepatocyte-specific functions such as albumin and AFP secretion. These findings provide strong evidence supporting the system’s potential for cell-based liver therapy.

### bFGF-loaded PLGA microspheres characterization

#### Physical properties and bFGF loading content

**Figure 3.**
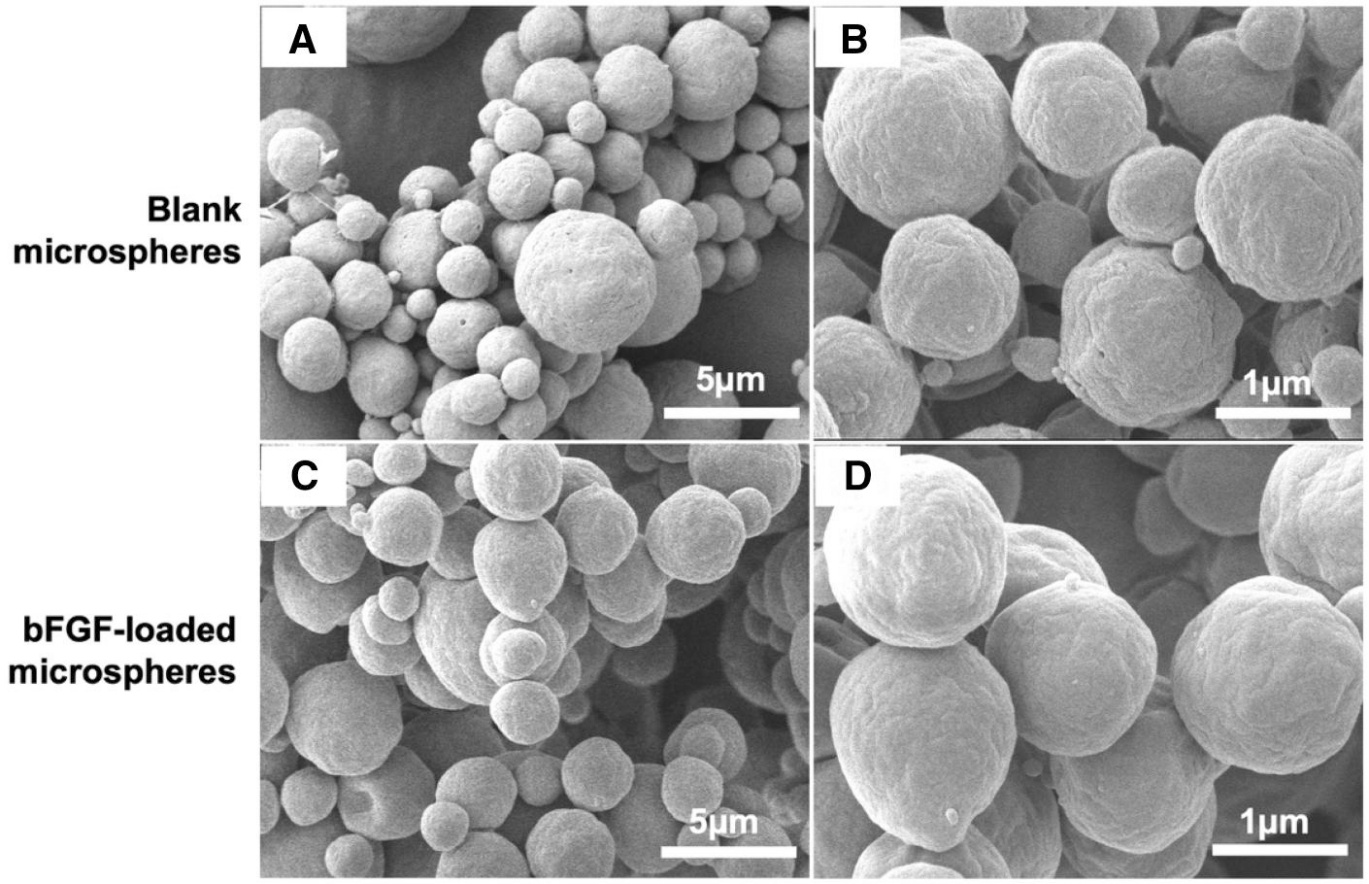
Representative SEM images of microsphere formulations at different magnifications. (**A**, **C**) show 4000× magnification views (scale bar is 5 µm), while (**B**, **D**) provide 12 000× magnification views (scale bar is 1 µm), revealing detailed surface morphology.

Unloaded and bFGF-loaded PLGA microspheres were successfully fabricated using the double emulsion solvent evaporation method (W_1_/O/W_2_). The mean size distribution, determined by DLS, was 3.34 ± 1.82 µm with a polydispersity index (PDI) of 0.05 ± 0.03 for unloaded microspheres and 4.25 ± 2.25 µm with a PDI of 0.08 ± 0.00 for bFGF-loaded microspheres. To further confirm microsphere size and homogeneity, SEM analysis was performed. The average size of unloaded PLGA microspheres, measured using ImageJ software, was 3.10 ± 0.87 µm, while bFGF-loaded microspheres measured 3.41 ± 0.87 µm. Encapsulation of bFGF did not significantly affect microsphere size or surface morphology, as both formulations exhibited a perfectly spherical shape with a smooth surface, as shown in Figure 3. The production yield was 24.42 ± 4.03% for unloaded microspheres and 26.67 ± 5.27% for bFGF-loaded microspheres. After purification to remove unencapsulated bFGF, the loading content, measured by ELISA, was (7.83 ± 0.60) × 10^−5^%, with an encapsulation efficiency of (35.23 ± 2.72) × 10^−2^%.

#### bFGF release kinetics

**Figure 4.**
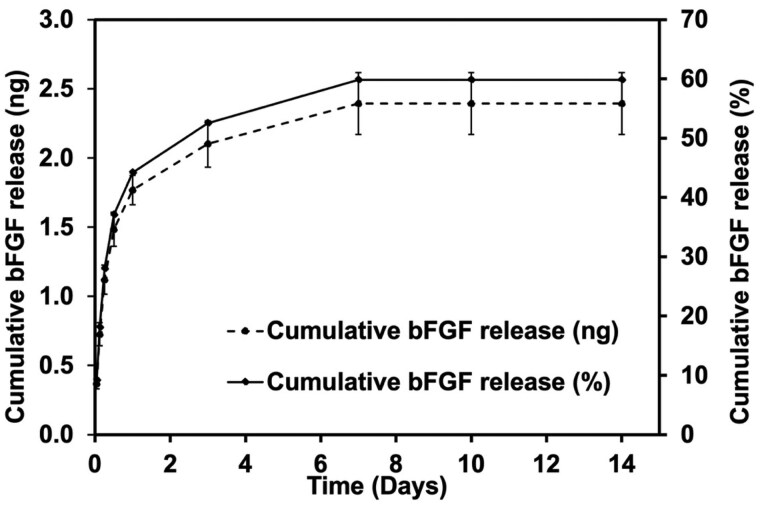
Cumulative release of bFGF over 14 days, presented in ng (left *y*-axis) and percentage release (right *y*-axis). A release phase is observed within the first 3 days, followed by a Plateau, with a total release of 2.39 ± 0.20 ng (59.83 ± 0.22%) by Day 14. Data are presented as mean ± standard deviation.

The cumulative release profile of bFGF from microspheres, presented in nanograms (ng) and percentage (%), is shown in Figure 4. The release profile demonstrated a biphasic pattern, with an initial burst phase followed by a sustained release over 14 days [33]. In the initial burst phase, 44% of bFGF (1.768 ng) was released within the first 48 h. The release then transitioned into a sustained phase, where bFGF continued to be released from the microspheres, reaching 59% (2.393 ng) by Day 7. A gradual release was maintained until Day 14, ensuring a prolonged supply of bFGF for encapsulated cells.

### ^1^H NMR characterization

The synthesis of PEG-tosyl was conducted using hydroxyl-terminated PEG as the starting material. The hydroxyl groups of PEG were selectively substituted with tosyl groups, which served as the preferred leaving group, facilitating subsequent functionalization. Firstly, the hydroxyl-terminated PEG was initiated by hydroxyl ions from the dissociation of NaOH. The tosyl group was conjugated with PEG after the addition of tosyl chloride, as shown in Supplementary Figure S1. After NaN_3_ was added, azide from NaN_3_ was replaced with tosyl in PEG. The peak of *f* = 3.33 ppm from the ^1^H NMR spectrum was indicated and used to characterize the azide group after the identical position of the tosyl group was removed. The percentage of the functional azide group was 72.71%, which was calculated by integrating the azide group compared with the theoretical azide group. The ^1^H NMR spectra of alginate and alkyne were presented in Supplementary Figure S2. The bond formation between alginate and alkyne was characterized by distinct peaks corresponding to propargylamine (*m* = 3.4 ppm) in Supplementary Figure S2 and the alginate alkyne group (*x* = 2.89 ppm), which confirms the successful conjugation of these components. ^1^H NMR spectra of alginate and RGD are shown in Supplementary Figure S3. The alginate-RGD spectrum shows successful conjugation, shown by the alkyne proton peak at 2.75 ppm (*x*) and the RGD peak at 1.7 ppm (*y*), indicating the integration of alkyne and RGD functionalities into the alginate backbone.

**Figure 5.**
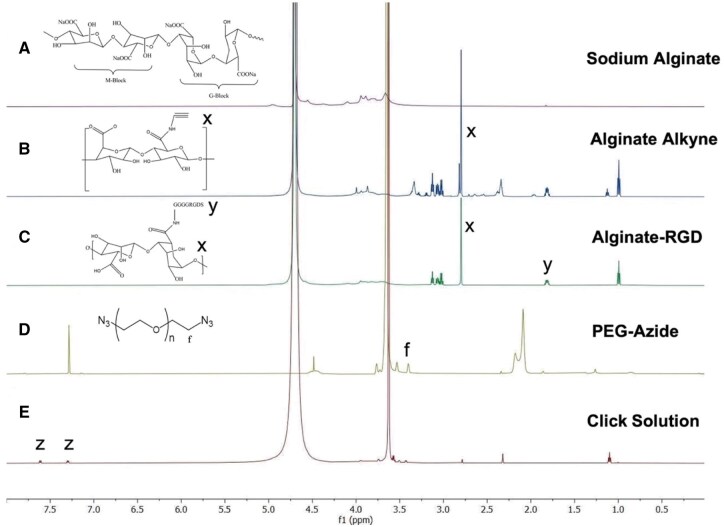
The successful functionalization of the click reaction was confirmed via ^1^H NMR spectroscopy, as evidenced by (**A**) the spectrum of sodium alginate shows its characteristic proton signals. (**B**) The distinct chemical shifts correspond to the alkyne group (*x* = 2.7 ppm). (**C**) The spectrum of alginate-RGD was confirmed by the detection of a characteristic RGD peak at 1.7 ppm (*y*) and an alginate-alkyne peak at 2.7 ppm (*x*) and (**D**) the azide group (*f* = 3.4 ppm). (**E**) The formation of two triazole rings via CuAAC, commonly referred to as the click reaction, at z = 7.3 and 7.6 ppm. The detailed results of ^1^H NMR are available in the Supplementary.

In the click reaction, alginate was functionalized by the addition of NHS and EDC as activating agents. These reagents facilitated the coupling between the alkyne group of alginate-alkyne and alginate-RGD with the azide group of PEG-azide. NHS and EDC were employed to activate the carboxyl groups of alginates, thereby promoting the formation of the desired covalent bonds. Following this, sodium ascorbate and CuSO_4_ were introduced to reduce Cu^2+^ to Cu^+^, which catalyzed the reaction between the alkyne groups of alginate-alkyne and alginate-RGD and the azide group from PEG-azide [34]. The reaction resulted in the formation of two 1,2,3-triazole rings, commonly referred to as the pentagonal bonds, which are characteristic products of the click reaction. In the ^1^H NMR spectrum, the alkyne group exhibited a signal at 2.7 ppm (*x*), while the azide group was observed at 3.75 ppm (*f*), as shown in Figure 5. The pentagonal bonds were characterized by signals at 7.25 and 7.75 ppm (*z*).

### Characterization of alginate-based click microbeads

#### Morphological analysis

**Figure 6.**
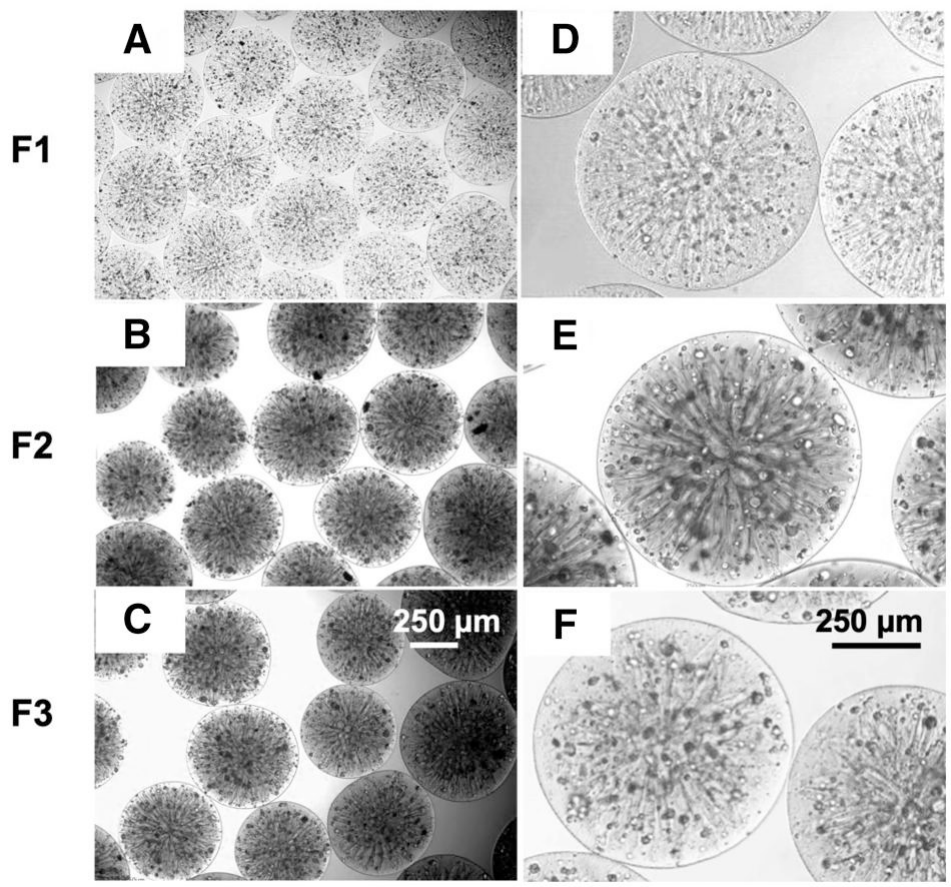
Representative bright-field images showing the size and morphology of three different alginate-based click microbead formulations. Microbeads in all formulations exhibit a consistent size distribution, as observed at 40× magnification (**A**–**C**). Higher magnification images (100×: **D**–**F**) reveal a flower-like structure inside the beads. Encapsulated cells are homogeneously distributed within the microbeads. The scale bar is 250 µm.

The encapsulation capacity and morphology of click-RGD microbeads are shown in Figure 6. Microbeads were fabricated using 1.5 mg/mL bFGF-loaded PLGA microspheres, 1 × 10^6^ cells/mL imHCs and 0.5 × 10^6^ cells/mL hMSCs. The fabricated microbeads exhibited a spherical shape and an estimated size range of 500–700 µm, as observed under an inverted microscope. A flower-like structure was observed inside the microbeads, indicating the formation of crosslinks from the click reaction. The microspheres were homogeneously distributed in size without any aggregation. The measured diameters of the different formulations were 402.58 ± 34.10 µm for F1, 460.24 ± 21.10 µm for F2 and 476.41 ± 43.28 µm for F3.

**Figure 7.**
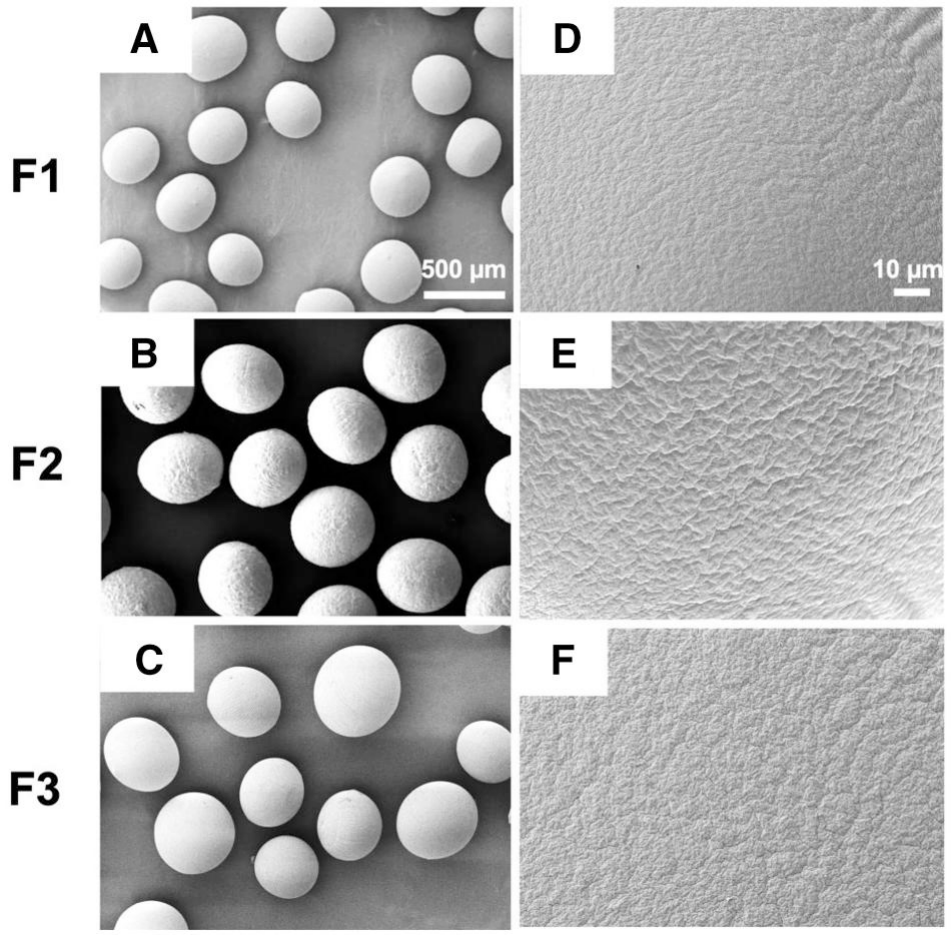
Representative SEM images of three different alginate-based click microbead formulations. Images were captured at 50× magnification (**A**–**C**) to observe overall structure and at 1000× magnification (**D**–**F**) to examine surface morphology. The scale bars are 500 and 10 µm.

SEM analysis revealed that F3 had the largest microbead size, followed by F 1 and F2, with measured diameters of 434.09 ± 22.68 μm, 402.28 ± 9.56 μm and 352.09 ± 11.54 μm, respectively. Additionally, all click-crosslinked formulations exhibited wrinkled surfaces, whereas the non-crosslinked beads maintained a smooth surface, as shown in Figure 7.

#### Assessment of structural integrity of microbeads

**Figure 8.**
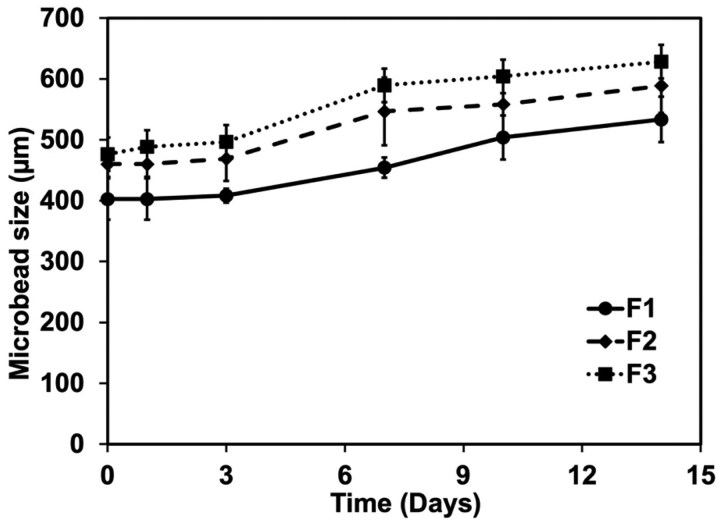
Structural integrity of microbead formulations (F1, F2 and F3) over 14 days, measured by changes in microbead size (µm). All formulations showed a gradual increase in size, with F2 and F3 exhibiting more significant expansion due to both swelling and increased cell proliferation. Data are presented as mean ± standard deviation.

Structural integrity of the microbeads in the cell culture medium was assessed by monitoring size changes over time in Figure 8. The results showed that for all formulations, bead size remained stable for the first three days. Afterward, the size gradually increased by 20% on Day 7 and by 30% on Day 14 compared to Day 0. The experiment was conducted over 14 days, after which the microbeads began to degrade. Among the formulations, F3 exhibited the largest size increase over time compared to F1 and F2. This may be attributed to the proliferation of encapsulated imHCs, enhanced by the coculturing effect of hMSCs and the presence of bFGF-loaded PLGA microspheres within the click-microbeads. Additionally, F1 was significantly smaller than F2 at all time points (*P* < 0.005). In contrast, the size of F2 and F3 remained consistent throughout the experiment.

#### Cell distribution and viability

**Figure 9.**
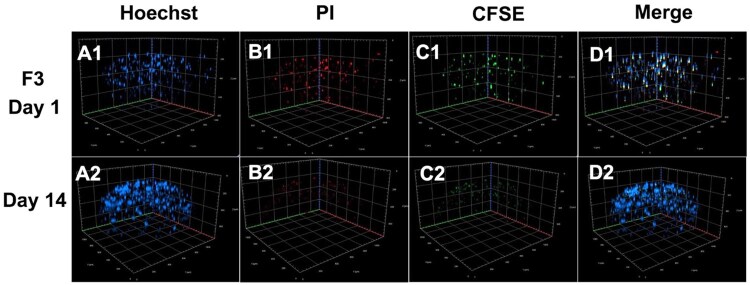
Representative 3D confocal microscopy images showing cell distribution within F3 microbeads encapsulating imHCs, hMSCs and bFGF-loaded PLGA microspheres on Day 1 (A1-D1) and Day 14 (A2-D2). Hoechst stains all nuclei, propidium iodide (PI) marks dead cells and CFSE labels live metabolically active hMSCs.

Cell distribution and viability within microbeads are critical parameters for evaluating the effectiveness of encapsulation systems in therapeutic applications. 3D confocal microscopy images of the spatial distribution and viability of encapsulated cells within F3 microbeads on Day 1 (A1-D1) and Day 14 (A2-D2) are shown in Figure 9. In contrast, corresponding images for F1 and F2 are provided in Figures S4 and S5, respectively, in the Supplementary. Hoechst staining (blue, A1-A2) confirmed the presence of nuclei throughout the microbeads, indicating even cell distribution. PI staining (red, B1-B2) revealed a few dead cells, with a slightly reduced signal on Days 1 and 14. CFSE staining (green, C1-C2) demonstrated intense metabolic activity in live hMSCs, which increased over time, suggesting active proliferation. Merged images (D1-D2) showed a predominance of blue and green fluorescence, confirming high viability and widespread distribution of cells. These findings indicate that the F3 effectively supports long-term cell survival and growth within the microbead structure. This result is consistent with fluorescent imaging, as shown in Supplementary Figure S6.

**Figure 10.**
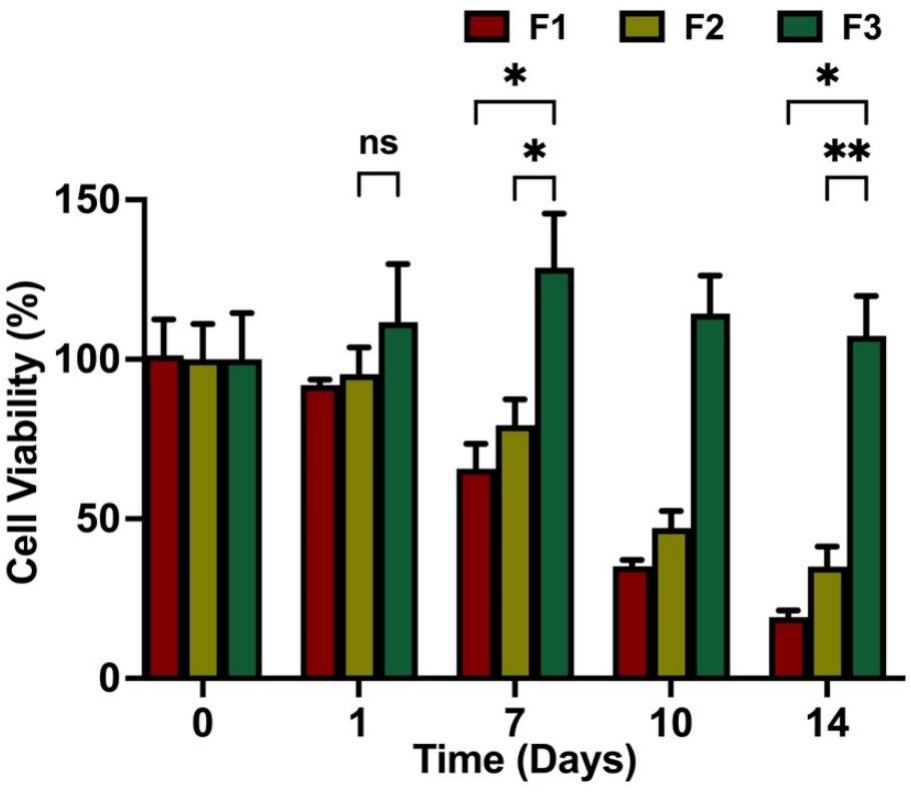
Cell viability (%) over 14 days for different microbead formulations (F1, F2 and F3). initially, all formulations exhibit high viability close to 100%. Data are presented as mean ± standard deviation.

The cell viability results illustrate distinct differences among the three formulations in Figure 10. At Day 0, all formulations exhibited similar cell viability, approaching 100%, as expected for freshly encapsulated cells, with subsequent normalization of cell viability at the following time points. By Day 1, a slight decrease is observed in F1 and F2, with viability around 91.95 ± 1.41 and 95.30 ± 6.88%. Conversely, cell viability in F3 exhibited a slight increase, reaching (112.00 ± 14.81%, *P* = 0.05). At Day 7, F3 demonstrates a significant increase, reaching approximately 129.00 ± 13.64%, suggesting that its composition provides a favorable environment for cell proliferation. In contrast, the viability of F1 and F2 declined to (65.70 ± 6.39, *P* < 0.05) and (79.30 ± 6.67%, *P* < 0.05), respectively, compared to F3. At Day 14, F3 retains the highest viability, 107.00 ± 10.27%, while F1 and F2 show markedly lower viability levels at (19.25 ± 1.67%, *P* < 0.05) and (35.07 ± 5.06%, *P* < 0.01), respectively.

The diminished CFSE signal intensity on Day 14 is likely due to progressive dye dilution through successive cell divisions, rather than a loss of cell viability. Notably, compared to the microbeads encapsulating hMSCs, imHCs and blank PLGA microspheres (F2), F3, which included bFGF-loaded PLGA microspheres, demonstrated higher stem cell viability. This is further supported by the increased number of Hoechst-positive nuclei, indicating enhanced cellular proliferation within the construct.

These findings suggest that F3 offers superior conditions for sustained cell viability and growth, potentially due to enhanced nutrient diffusion, biocompatibility or structural properties that better support cell survival. Moreover, sustained release of bFGF in F3 played a key role in promoting cell growth and survival, providing essential growth factors that are crucial for hepatocyte proliferation and maintenance.

The observed enhancement in cell viability in F3 is presumed to result from the synergistic interaction of multiple components within the system. These include integrin-mediated cell-matrix interactions facilitated by RGD functionalization, enhanced network stability conferred by PEG crosslinking, sustained trophic support via controlled bFGF release and paracrine effects derived from co-encapsulated hMSCs. While this study did not aim to isolate the contribution of each factor, the findings collectively highlight the efficacy of this integrative strategy in creating a bioactive microenvironment conducive to encapsulated cellular viability and function.

#### Functionality of encapsulated imHCs

**Figure 11.**
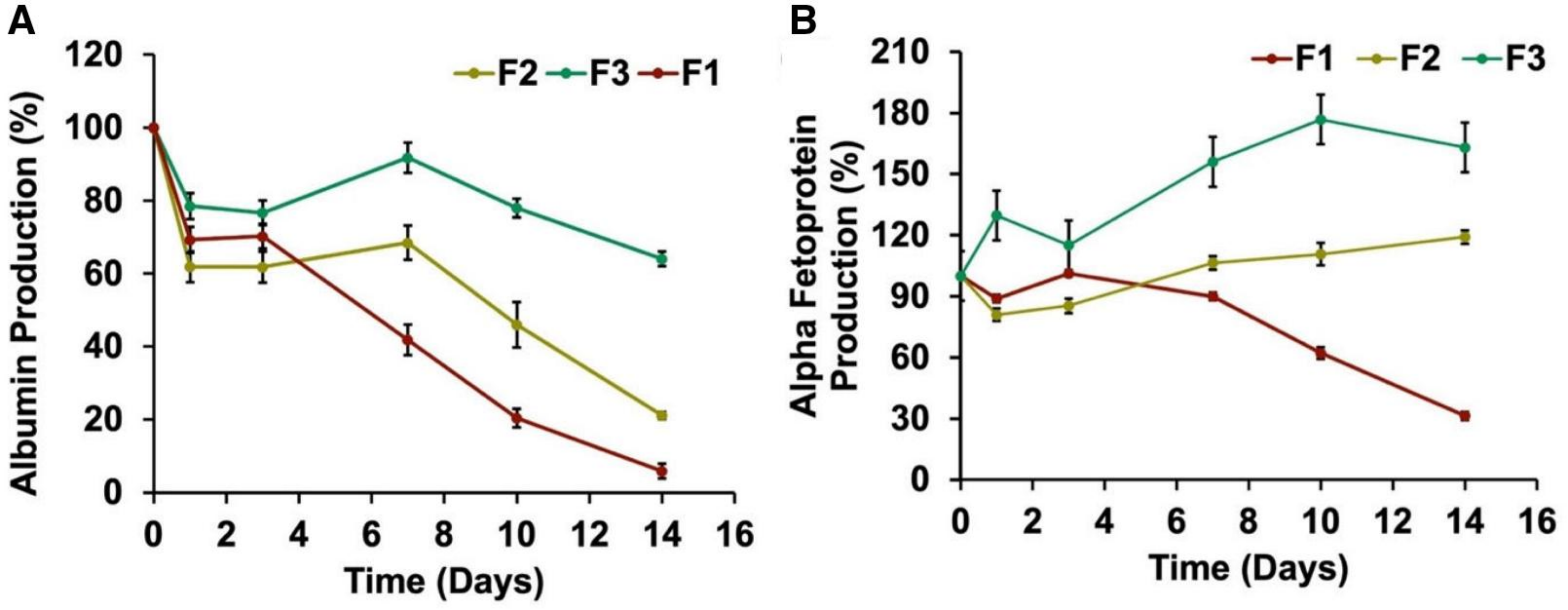
Albumin and AFP secretion (%) over 14 days in different microbead formulations. (**A**) Albumin secretion (%) decreased over time for F1 and F2, whereas F3 maintained higher levels, particularly on Days 10 and 14. (**B**) AFP secretion (%) remained stable in the early days but increased significantly in F3 by Day 10 and was maintained by Day 14. Data are presented as mean ± standard deviation.

To evaluate the hepatic functionality of encapsulated imHCs, albumin and AFP secretion were quantified using ELISA over 14 days. All formulations exhibited high initial albumin secretion on Day 0 (3200–3800 ng), which declined over time. By Day 7, F3 retained significantly higher albumin levels (2910.53 ± 74.74 ng) compared to F2 (2475.27 ± 206.31 ng) and F1 (1574.80 ± 6.37 ng). This trend continued through Day 14, where F3 maintained albumin secretion (2032.53 ± 29.45 ng), which was significantly higher than both F2 (761.80 ± 6.21 ng) and F1 (221.87 ± 14.01 ng) in Supplementary Figure S7A. The albumin production trends for three formulations were evaluated with Day 0 values normalized to 100% in Figure 11. On Day 7, F3 demonstrated the highest albumin production, retaining 91.70 ± 4.18% of its Day 0 secretion levels, followed by F2 at 68.40 ± 4.64% (*P* = 0.05) and F1 at 41.90 ± 0.65% (*P* < 0.01). On Day 14, F3 maintained 64.00 ± 2.00% of its Day 0 production, while F2 decreased to 21.10 ± 1.02% (*P* < 0.0001) and F1 showed the most significant reduction, with only 5.90 ± 0.39% (*P* < 0.0001) of its initial albumin production remaining in Figure 11A. These results indicate that F3 exhibited the most sustained albumin secretion over the 14 days, while F2 and F1 showed progressively more significant declines in production.

Similarly, AFP secretion in F3 increased to 1146.00 ± 13.90 ng by Day 10 and remained maintained on Day 14 (1057.00 ± 9.19 ng), significantly higher than F2 (976.00 ± 3.90 ng) and F1 (302.00 ± 20.06 ng) in Supplementary Figure S7B. F3 exhibited the highest AFP production percentage on Day 14, as shown in Figure 11B, with a value of 163.06 ± 1.50%, indicating sustained secretion. F2 demonstrated a more moderate response, maintaining 119.15 ± 3.34% (*P* < 0.05), while F1 showed the lowest AFP production, with a value of 31.35 ± 2.00% (*P* < 0.0001), indicating a significant decline in secretion over the 14 days. The results showed that F3 exhibited the highest and most sustained AFP production over the 14 days, while F1 demonstrated a marked reduction in secretion, highlighting significant differences in the formulations’ capacities to sustain AFP production.

The sustained albumin and AFP production in F3 showed superior hepatocyte viability and differentiation. In contrast, F1 and F2 displayed significantly lower functional outputs, underscoring the critical importance of an integrated bio-functional microenvironment for long-term hepatic support. The sustained albumin production observed in F3 reflects mature hepatocyte activity, while the elevated AFP levels indicate ongoing hepatic differentiation. These findings suggest that F3 most effectively supported hepatocyte viability and functional maturation, providing an optimal microenvironment for the function of imHCs. In contrast, the lower functionality observed in F1 and F2 highlights the limitations of unmodified or partially modified alginate microbeads, which may not provide sufficient support for sustained hepatocyte differentiation and function.

## Discussion

ALF remains a life-threatening condition with limited therapeutic options beyond liver transplantation. However, the shortage of donor organs, surgical risks and complications associated with long-term immunosuppression necessitate the development of alternative treatment strategies. Cell-based therapies, particularly hepatocyte encapsulation, have emerged as promising approaches for providing temporary liver support while promoting regeneration. Yet, conventional alginate-based systems suffer from poor structural integrity, a lack of cell adhesion and insufficient biochemical signaling to sustain hepatocyte viability and function. To address these limitations, we developed a multifunctional microencapsulation platform based on click-RGD-modified alginate microbeads, co-encapsulating bFGF-loaded PLGA microspheres, imHCs and hMSCs [34].

In our previous investigation, co-encapsulation of imHCs and bFGF-loaded PLGA microspheres within unmodified alginate microbeads demonstrated limited therapeutic potential [11]. Although initial cell viability was maintained, the absence of extracellular matrix-mimetic cues and bioactive adhesion motifs resulted in poor cell-matrix interactions, contributing to a marked decline in hepatocyte function over time. By Day 14, albumin secretion had significantly decreased and cell viability fell below 40%. Furthermore, the unmodified alginate matrix exhibited considerable swelling, compromising microbead structural integrity and potentially disrupting sustained bFGF release. These limitations provided the rationale for the present study, in which we employed a click-functionalized alginate microbead system modified with RGD peptides and PEG cross-linking. Additionally, hMSCs were co-encapsulated with imHCs and bFGF-loaded PLGA microspheres to enhance paracrine support, promote immunomodulation and improve overall hepatocyte viability and function within the 3D microenvironment.

Prior studies in the literature have shown that RGD modification improves cell spreading and proliferation in both 2D and 3D alginate systems [35]. Similarly, PEG, a biocompatible and FDA-approved polymer, was incorporated via click chemistry to improve the microbead’s structural integrity [36]. PEG crosslinking mitigates degradation caused by cation leakage and enhances hydrogel stress relaxation, thereby supporting long-term cell survival and proliferation [37].

Microbeads were fabricated using electrostatic and vibrating-jet extrusion techniques, which enabled the production of uniform, spherical microbeads around 500 µm that are suitable for intraperitoneal injection [38–42]. Optimization of key parameters, including vibration frequency, flow rate, nozzle size, voltage and stirring speed, allowed precise control of bead size and morphology [43]. Structural integrity testing in the culture medium confirmed that PEG-crosslinked beads remained structurally intact for over 14 days. Furthermore, SEM confirmed adequate porosity to allow diffusion of cytokines and metabolic waste, ensuring a supportive microenvironment for encapsulated cells.

The current bFGF loading content was (7.83 ± 0.60) × 10^−5^%, with an encapsulation efficiency of (35.23 ± 2.72) × 10^−2^%, which was suboptimal, consistent with known challenges in encapsulating hydrophilic proteins via double emulsion methods. Despite the low encapsulation efficiency, the amount of loaded bFGF was sufficient to maintain hepatocyte viability and functionality in our system. PLGA was chosen due to its established capability to provide sustained protein release over several days.

To improve loading efficiency, previous studies suggest incorporating heparin as a stabilizing agent, given its strong electrostatic interaction with bFGF. This interaction effectively minimizes premature diffusion and enhances bFGF retention within the PLGA microsphere matrix [44]. Additionally, bovine serum albumin (BSA) serves as a stabilizing excipient, protecting bFGF from interfacial denaturation and improving encapsulation efficiency. Co-lyophilization of bFGF with heparin and BSA further enhances protein stability during emulsification. Process modifications, such as minimizing the internal aqueous phase volume and reducing shear exposure, may also reduce protein loss. Adopting these approaches can significantly improve bFGF encapsulation while preserving bioactivity for sustained-release applications.

These fabrication and material choices directly contributed to the observed enhancement in cell viability and hepatic function. Click-RGD microbeads provided improved mechanical robustness and cellular support compared to unmodified alginate beads [11]. Encapsulated imHCs demonstrated prolonged secretion of albumin and AFP markers, indicative of both hepatic maturity and early differentiation stages, respectively. Albumin reflects hepatocyte-specific metabolic function, while AFP is associated with fetal hepatocyte-like phenotypes [45]. The co-expression of these markers suggests that the encapsulation environment effectively supports progressive hepatocyte differentiation over time. In addition, the co-encapsulation of hMSCs further enhanced cell viability and function, likely through paracrine signaling and immunomodulatory effects. These findings align with previous studies demonstrating that hMSCs contribute to tissue repair and immune modulation, mainly when used in conjunction with hepatocyte transplantation strategies [46–48].

The results of this study demonstrated that the F3, consisting of hMSCs, imHCs and bFGF-loaded PLGA microspheres, exhibited superior performance in terms of sustained hepatocyte function, as evidenced by the prolonged secretion of albumin and AFP over 14 days. This contrasted with F1 and F2, which showed reduced hepatocyte function over time. These factors contributed to an optimal microenvironment that supported hepatocyte differentiation, survival and maturation, as indicated by the sustained production of both albumin and AFP, which are markers indicative of hepatocyte maturity and early stages of differentiation, respectively [45].

Despite these promising outcomes, several limitations remain. Optimization of extrusion parameters and crosslinking conditions may further improve microbead uniformity and encapsulation efficiency. The ideal bFGF loading concentration and cell seeding density must also be optimized to prevent overcrowding or nutrient depletion. Moreover, this study was limited to *in vitro* evaluation; thus, future studies should focus on *in vivo* validation, including assessments of immune response, long-term structural integrity and therapeutic efficacy.

## Conclusion

This study successfully developed a click-RGD functionalized microbead system for hepatocyte encapsulation, integrating bFGF-loaded PLGA microspheres, imHCs and hMSCs to enhance cell viability, proliferation and liver-specific function. The results demonstrated that the click-RGD modification improved cell adhesion, while controlled bFGF release sustained hepatocyte survival and function, leading to prolonged albumin and AFP secretion. In this study, F3 exhibited superior performance in supporting long-term hepatocyte differentiation and viability, with enhanced albumin and AFP secretion over 14 days. Therefore, the co-encapsulation of hMSCs further enhanced hepatocyte viability, highlighting the potential of this system for regenerative liver therapies. By combining mechanical reinforcement, biochemical support and sustained growth factor delivery, this encapsulation platform addresses key limitations of conventional alginate microbeads and provides a promising, scalable and minimally invasive alternative to liver transplantation. Future *in vivo* studies will be essential to evaluate long-term functionality and clinical translation [49]. Future studies will focus on validating the therapeutic efficacy of the encapsulated hepatocytes *in vivo* using a rat model of ALF, aiming to bridge the gap toward clinical translation. Overall, this study represents a significant step forward in cell-based liver therapies, offering a novel strategy to support hepatocyte transplantation and liver regeneration in patients with ALF.

## Funding

This research project has been supported by Mahidol University (Fundamental Fund: fiscal year 2025 by National Science Research and Innovation Fund). The first author was supported by the Thailand Research Fund and the Thailand International Cooperation Agency through the Royal Golden Jubilee Ph.D. Program under Grant Ph.D./0052/2561.

## Supplementary Material

rbaf094_Supplementary_Data

## References

[1] Wu Z , Han M, Chen T, Yan W, Ning Q. Acute liver failure: mechanisms of immune‐mediated liver injury. Liver Int 2010;30:782–94. 20492514 10.1111/j.1478-3231.2010.02262.x

[2] Jitraruch S , Dhawan A, Hughes RD, Filippi C, Soong D, Philippeos C, Lehec SC, Heaton ND, Longhi MS, Mitry RR. Alginate microencapsulated hepatocytes optimised for transplantation in acute liver failure. PLoS One 2014;9:e113609. 25438038 10.1371/journal.pone.0113609 PMC4249959

[3] Orive G , Santos E, Poncelet D, Hernández RM, Pedraz JL, Wahlberg LU, De Vos P, Emerich D. Cell encapsulation: technical and clinical advances. Trends Pharmacol Sci 2015;36:537–46. 26067102 10.1016/j.tips.2015.05.003

[4] Gasperini L , Mano JF, Reis RL. Natural polymers for the microencapsulation of cells. J R Soc Interface 2014;11:20140817. 25232055 10.1098/rsif.2014.0817 PMC4191114

[5] Samadi A , Moammeri A, Azimi S, Bustillo-Perez BM, Mohammadi MR. Biomaterial engineering for cell transplantation. Biomater Adv 2024;158:213775. 38252986 10.1016/j.bioadv.2024.213775

[6] Karaca MA , Kancagi DD, Ozbek U, Ovali E, Gok O. Preparation of Cell-Loaded microbeads as stable and injectable delivery platforms for tissue engineering. Biomimetics 2023;8:155. 37092407 10.3390/biomimetics8020155 PMC10123749

[7] Abbas SEM , Maged G, Wang H, Lotfy A. Mesenchymal stem/stromal cells microencapsulation for cell therapy. Cells 2025;14:149. 39936941 10.3390/cells14030149 PMC11817150

[8] Liu W-H , Song F-Q, Ren L-N, Guo W-Q, Wang T, Feng Y-X, Tang L-J, Li K. The multiple functional roles of mesenchymal stem cells in participating in treating liver diseases. J Cell Mol Med 2015;19:511–20. 25534251 10.1111/jcmm.12482 PMC4369809

[9] Tsuchiya A , Takeuchi S, Watanabe T, Yoshida T, Nojiri S, Ogawa M, Terai S. Mesenchymal stem cell therapies for liver cirrhosis: MSCs as “conducting cells” for improvement of liver fibrosis and regeneration. Inflamm Regen 2019;39:18–6. 31516638 10.1186/s41232-019-0107-z PMC6732839

[10] Raus RA , Nawawi WMFW, Nasaruddin RR. Alginate and alginate composites for biomedical applications. Asian J Pharm Sci 2021;16:280–306. 34276819 10.1016/j.ajps.2020.10.001 PMC8261255

[11] Saimok W , Iyaraganjanakul P, Kreeporn P, Phuanghom W, Sa-ngiamsuntorn K, Hongeng S, Nasongkla N. Co-encapsulation of bFGF-loaded microspheres and hepatocytes in microbeads for prolonging hepatic pre-transplantation. J Drug Deliv Sci Technol 2023;87:104784.

[12] Win SY , Chavalitsarot M, Eawsakul K, Ongtanasup T, Nasongkla N. Encapsulation of cyclosporine A-Loaded PLGA nanospheres in alginate microbeads for anti-inflammatory application. ACS Omega 2024;9:6901–11. 38371838 10.1021/acsomega.3c08438 PMC10870416

[13] Colombo M , Bianchi A. Click chemistry for the synthesis of RGD-containing integrin ligands. Molecules 2010;15:178–97. 20110882 10.3390/molecules15010178 PMC6256992

[14] Ma TY , Kikuchi M, Sarfeh IJ, Shimada H, Hoa NT, Tarnawski AS. Basic fibroblast growth factor stimulates repair of wounded hepatocyte monolayer: modulatory role of protein kinase a and extracellular matrix. J Lab Clin Med 1999;134:363–71. 10521082 10.1016/s0022-2143(99)90150-6

[15] Lee H , Cusick RA, Browne F, Ho Kim T, Ma PX, Utsunomiya H, Langer R, Vacanti JP. Local delivery of basic fibroblast growth factor increases both angiogenesis and engraftment of hepatocytes in tissue-engineered polymer devices. Transplantation 2002;73:1589–93. 12042644 10.1097/00007890-200205270-00011

[16] Wang Y-H , Wu D-B, Chen B, Chen E-Q, Tang H. Progress in mesenchymal stem cell–based therapy for acute liver failure. Stem Cell Res Ther 2018;9:227–9. 30143052 10.1186/s13287-018-0972-4 PMC6109312

[17] Leventhal A , Chen G, Negro A, Boehm M. The benefits and risks of stem cell technology. Oral Dis 2012;18:217–22. 22093062 10.1111/j.1601-0825.2011.01870.x PMC3752464

[18] Giri TK , Choudhary C, Alexander A, Badwaik H, Tripathi DK, Ajazuddin.,. Prospects of pharmaceuticals and biopharmaceuticals loaded microparticles prepared by the double emulsion technique for controlled delivery. Saudi Pharm J 2013;21:125–41. 23960828 10.1016/j.jsps.2012.05.009 PMC3744931

[19] Horprasertkij K , Dwivedi A, Riansuwan K, Kiratisin P, Nasongkla N. Spray coating of dual antibiotic-loaded nanospheres on orthopedic implant for prolonged release and enhanced antibacterial activity. J Drug Deliv Sci Technol 2019;53:101102.

[20] Caputo TM , Barisciano G, Mulè C, Cusano AM, Aliberti A, Muccillo L, Colantuoni V, Sabatino L, Cusano A. Development of high-loading trastuzumab PLGA nanoparticles: a powerful tool against HER2-positive breast cancer cells. Int J Nanomedicine 2023;18:6999–7020. 38034948 10.2147/IJN.S429898 PMC10683664

[21] Srisang S , Nasongkla N. Spray coating of a Foley urinary catheter by chlorhexidine-loadedpoly (ε-caprolactone) nanospheres: effect of lyoprotectants, characteristics, and antibacterial activity evaluation. Pharm Dev Technol 2019;24:402–9. 30265590 10.1080/10837450.2018.1502317

[22] Vanni S , Caputo TM, Cusano AM, De Vita A, Cusano A, Cocchi C, Mulè C, Principe S, Liverani C, Celetti G, Micco A, Spadazzi C, Miserocchi G, Ibrahim T, Mercatali L, Aliberti A. Engineered anti-HER2 drug delivery nanosystems for the treatment of breast cancer. Nanoscale 2025;17:9436–57. 40116284 10.1039/d4nr03907f

[23] Chinavinijkul P , Riansuwan K, Kiratisin P, Srisang S, Nasongkla N. Dip-and spray-coating of schanz pin with PLA and PLA nanosphere for prolonged antibacterial activity. J Drug Deliv Sci Technol 2021;65:102667.

[24] Sandvig I , Karstensen K, Rokstad AM, Aachmann FL, Formo K, Sandvig A, Skjåk-Bræk G, Strand BL. RGD‐peptide modified alginate by a chemoenzymatic strategy for tissue engineering applications. J Biomed Mater Res A 2015;103:896–906. 24826938 10.1002/jbm.a.35230

[25] Kaklamani G , Kazaryan D, Bowen J, Iacovella F, Anastasiadis SH, Deligeorgis G. On the electrical conductivity of alginate hydrogels. Regen Biomater 2018;5:293–301. 30338127 10.1093/rb/rby019 PMC6184632

[26] Mohammadalinejhad S , Almonaitytė A, Jensen I-J, Kurek M, Lerfall J. Alginate microbeads incorporated with anthocyanins from purple corn (*Zea mays* L.) using electrostatic extrusion: microencapsulation optimization, characterization, and stability studies. Int J Biol Macromol 2023;246:125684. 37406909 10.1016/j.ijbiomac.2023.125684

[27] Omer AM , Elmeligy MA, Abd El-Monaem EM, Naiel BH, Barlog M, Heydari A. pH-sensitive aminated chitosan/carboxymethyl cellulose/aminated graphene oxide coated composite microbeads for efficient encapsulation and sustained release of 5-fluorouracil. Int J Biol Macromol 2024;283:137250. 39522920 10.1016/j.ijbiomac.2024.137250

[28] Çakmak E. Fabrication of silver nanoparticles decorated on sodium alginate microbeads enriched with keratin and investigation of its catalytic and antioxidant activity. Int J Biol Macromol 2024;267:131478. 38604434 10.1016/j.ijbiomac.2024.131478

[29] Chen Jianwei , Liu Zeyang, Wang Zixian, Zhang Xiuxiu, Zhang Yi, Zhan Zhen, Gong Xiaohua, Xu Tao. One-step biofabrication of liquid core—GelMa shell microbeads for in situ hollow cell ball self-assembly. Regen Biomater 2024;11:rbae021. 38525324 10.1093/rb/rbae021 PMC10960924

[30] Wang Zixian , Zhang Xiuxiu, Xue Limin, Wang Gangwei, Li Xinda, Chen Jianwei, Xu Ruxiang, Xu Tao. A controllable gelatin-based microcarriers fabrication system for the whole procedures of MSCs amplification and tissue engineering. Regen Biomater 2023;10:rbad068. 37638061 10.1093/rb/rbad068 PMC10458456

[31] Shi X-L , Zhang Y, Gu J-Y, Ding Y-T. Coencapsulation of hepatocytes with bone marrow mesenchymal stem cells improves hepatocyte-specific functions. Transplantation 2009;88:1178–85. 19935371 10.1097/TP.0b013e3181bc288b

[32] Eawsakul K , Tancharoen S, Nasongkla N. Combination of dip coating of BMP-2 and spray coating of PLGA on dental implants for osseointegration. J Drug Deliv Sci Technol 2021;61:102296.

[33] Basmanav FB , Kose GT, Hasirci V. Sequential growth factor delivery from complexed microspheres for bone tissue engineering. Biomaterials 2008;29:4195–204. 18691753 10.1016/j.biomaterials.2008.07.017

[34] Fraczyk J , Wasko J, Walczak M, Kaminski ZJ, Puchowicz D, Kaminska I, Bogun M, Kolasa M, Stodolak-Zych E, Scislowska-Czarnecka A, Kolesinska B. Conjugates of copper alginate with arginine-glycine-aspartic acid (RGD) for potential use in regenerative medicine. Materials 2020;13:337. 31940765 10.3390/ma13020337 PMC7013949

[35] Dumbleton J , Agarwal P, Huang H, Hogrebe N, Han R, Gooch KJ, He X. The effect of RGD peptide on 2D and miniaturized 3D culture of HEPM cells, MSCs, and ADSCs with alginate hydrogel. Cell Mol Bioeng 2016;9:277–88. 27990180 10.1007/s12195-016-0428-9 PMC5157694

[36] Gattás-Asfura KM , Stabler CL. Chemoselective cross-linking and functionalization of alginate via staudinger ligation. Biomacromolecules 2009;10:3122–9. 19848408 10.1021/bm900789a PMC2996242

[37] Vieira S , Silva‐Correia J, Reis RL, Oliveira JM. Engineering hydrogels for modulation of material‐cell interactions. Macromol Biosci 2022;22:e2200091. 35853666 10.1002/mabi.202200091

[38] Dhawan A , Chaijitraruch N, Fitzpatrick E, Bansal S, Filippi C, Lehec SC, Heaton ND, Kane P, Verma A, Hughes RD, Mitry RR. Alginate microencapsulated human hepatocytes for the treatment of acute liver failure in children. J Hepatol 2020;72:877–84. 31843649 10.1016/j.jhep.2019.12.002

[39] Rana D , Tabasum A, Ramalingam M. Cell-laden alginate/polyacrylamide beads as carriers for stem cell delivery: preparation and characterization. RSC Adv 2016;6:20475–84.

[40] Gasperini L , Maniglio D, Migliaresi C. Microencapsulation of cells in alginate through an electrohydrodynamic process. J Bioact Compat Polym 2013;28:413–25.

[41] Mazzitelli S , Tosi A, Balestra C, Nastruzzi C, Luca G, Mancuso F, Calafiore R, Calvitti M. Production and characterization of alginate microcapsules produced by a vibrational encapsulation device. J Biomater Appl 2008;23:123–45. 18467747 10.1177/0885328207084958

[42] Whelehan M , Marison IW. Microencapsulation using vibrating technology. J Microencapsul 2011;28:669–88. 22047545 10.3109/02652048.2011.586068

[43] Lengyel M , Balogh E, Szerőczei D, Dobó-Nagy C, Pápay Z, Stömmer V, Klebovich I, Antal I. Study on process parameters and optimization of microencapsulation based on phase separation. Eur J Pharm Sci 2018;122:273–80. 29981890 10.1016/j.ejps.2018.07.015

[44] Si H-B , Zeng Y, Lu Y-R, Cheng J-Q, Shen B. Control-released basic fibroblast growth factor-loaded poly-lactic-co-glycolic acid microspheres promote sciatic nerve regeneration in rats. Exp Ther Med 2017;13:429–36. 28352311 10.3892/etm.2016.4013 PMC5348676

[45] Baxter M , Withey S, Harrison S, Segeritz C-P, Zhang F, Atkinson-Dell R, Rowe C, Gerrard DT, Sison-Young R, Jenkins R, Henry J, Berry AA, Mohamet L, Best M, Fenwick SW, Malik H, Kitteringham NR, Goldring CE, Piper Hanley K, Vallier L, Hanley NA. Phenotypic and functional analyses show stem cell-derived hepatocyte-like cells better mimic fetal rather than adult hepatocytes. J Hepatol 2015;62:581–9. 25457200 10.1016/j.jhep.2014.10.016 PMC4334496

[46] Liu P , Qian Y, Liu X, Zhu X, Zhang X, Lv Y, Xiang J. Immunomodulatory role of mesenchymal stem cell therapy in liver fibrosis. Front Immunol 2022;13:1096402. 36685534 10.3389/fimmu.2022.1096402 PMC9848585

[47] Wen F , Yang G, Yu S, Liu H, Liao N, Liu Z. Mesenchymal stem cell therapy for liver transplantation: clinical progress and immunomodulatory properties. Stem Cell Res Ther 2024;15:320. 39334441 10.1186/s13287-024-03943-6 PMC11438256

[48] Yang Y , Zhao Y, Zhang L, Zhang F, Li L. The application of mesenchymal stem cells in the treatment of liver diseases: mechanism, efficacy, and safety issues. Front Med (Lausanne) 2021;8:655268. 34136500 10.3389/fmed.2021.655268 PMC8200416

[49] Yagi S , Hirata M, Miyachi Y, Uemoto S. Liver regeneration after hepatectomy and partial liver transplantation. Int J Mol Sci 2020;21:8414. 33182515 10.3390/ijms21218414 PMC7665117

