# Supplementary material for: Co-encapsulation of hepatocytes, mesenchymal stem cells and growth factor in arginine-glycine-aspartate functionalized microbeads for liver disease
Source: Regen Biomater. 2025 Sep 16;12:rbaf094. doi: 10.1093/rb/rbaf094 (PMC12478700; doi:10.1093/rb/rbaf094)
Supplement: rbaf094_Supplementary_Data [file rbaf094_supplementary_data.docx]

**Supplementary Material**

**Co-Encapsulation of Hepatocytes, Mesenchymal Stem Cells, and Growth Factor in Arginine-Glycine-Aspartate Functionalized Microbeads for Liver Disease**

Su Yee Win^1,†^, Pinunta Nittayacharn^1,†^, Arkhom Saingam^2^, Khanit Sa-ngiamsuntorn^3^ and Norased Nasongkla^1,*^

^1^ Department of Biomedical Engineering, Faculty of Engineering, Mahidol University, Nakhon Pathom, 73170, Thailand

^2^ Cryoviva (Thailand) Co., Ltd., Khunkaew Nakhonchaisri, Nakhon Pathom 73120, Thailand

^3^ Department of Biochemistry, Faculty of Pharmacy, Mahidol University, Bangkok, 10400, Thailand

**
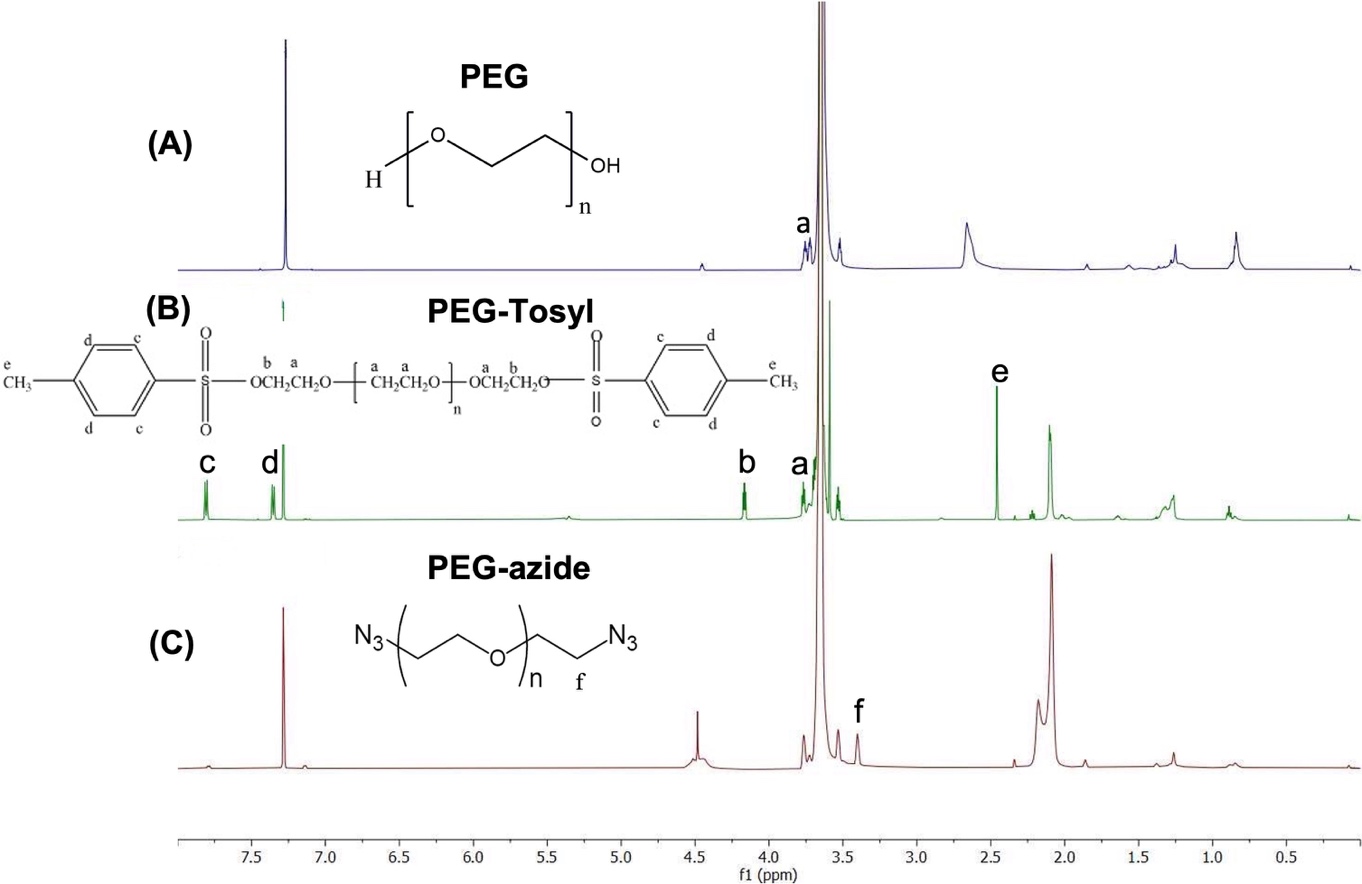
1. ^1^H NMR Characterization of PEG-Tosyl and PEG-Azide**

**Figure S1.** ^1^H NMR spectra confirming the stepwise synthesis of PEG-tosyl and PEG-azide derivatives. (A) Unmodified PEG exhibits the characteristic backbone proton signal (a) at 3.6 ppm. (B) PEG-tosyl displays new peaks corresponding to the methylene protons adjacent to the tosyl group (b) at 4.14 ppm and aromatic protons of the tosyl ring at 7.78 ppm (c), 7.3 ppm (d), and 2.43 ppm (e) (functionalization efficiency: 74.22%). (C) The PEG-azide spectrum exhibits the disappearance of tosyl-specific peaks (c, d) and the emergence of an azide-associated peak (f) at 3.33 ppm (functionalization efficiency: 72.71%).


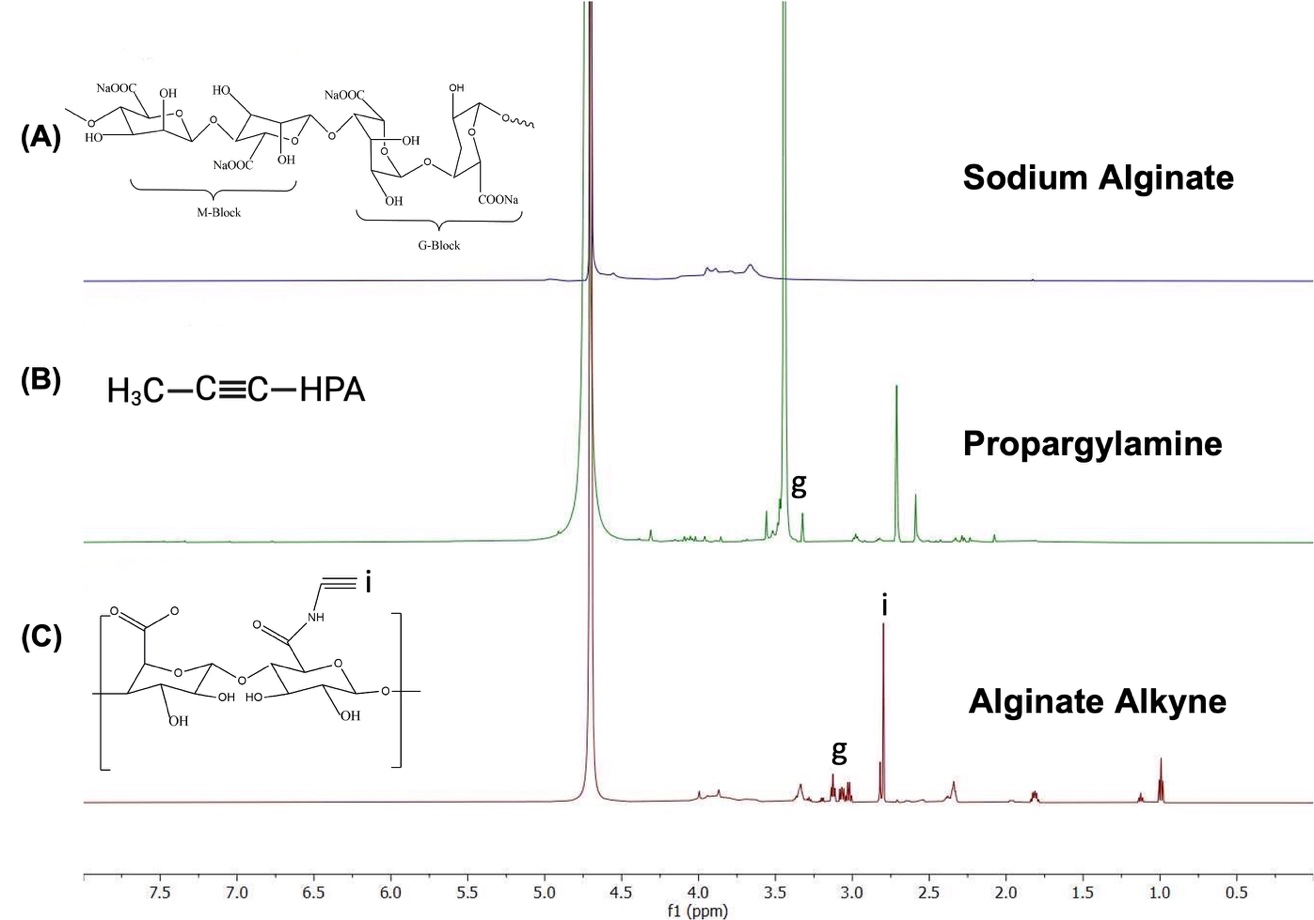
**2. ^1^H NMR Characterization of Alginate-Alkyne**

**Figure S2.** ^1^H NMR spectra confirming the successful conjugation of alkyne functionality to alginate via amide bond formation. (A) Sodium alginate displays its characteristic polysaccharide proton signals. (B) Propargylamine shows a distinct peak at 3.40 ppm (m), corresponding to methylene protons adjacent to the terminal alkyne. (C) The alginate-alkyne spectrum reveals a new peak at 2.89 ppm (x), assigned to the terminal alkyne proton.


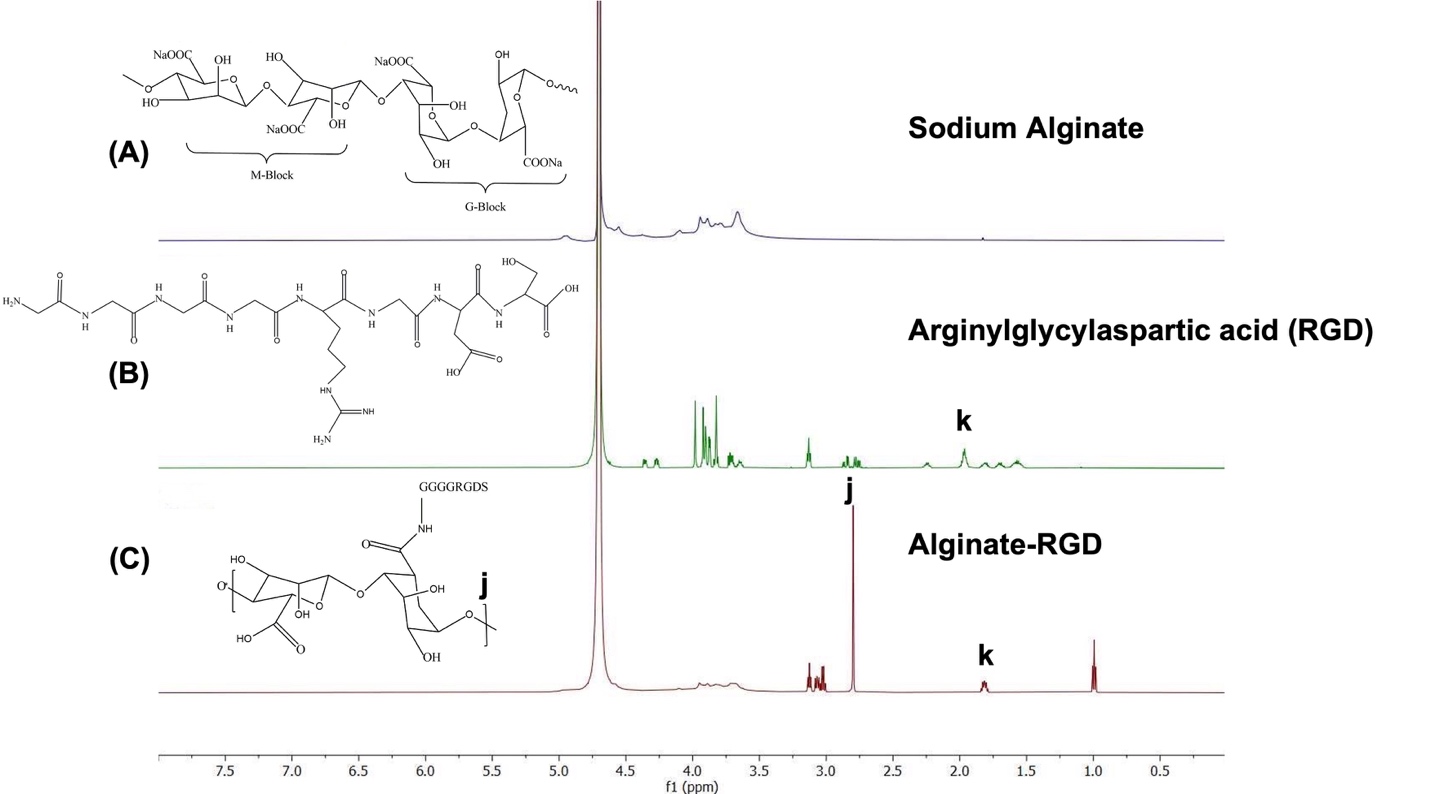
**3. ^1^H NMR Characterization of Alginate-RGD**

**Figure S3**. ^1^ H NMR spectra confirming dual functionalization of alginate with alkyne and RGD moieties via amide bond formation. (A) Sodium alginate shows characteristic polysaccharide proton signals. (B) The RGD peptide displays a peak at 1.8 ppm (y), corresponding to aliphatic side chains. (C) The alginate-RGD spectrum reveals new peaks at 2.75 ppm (x, terminal alkyne proton) and 1.7 ppm (y, RGD-associated protons).

**4. Assessment of Cell Viability and Distribution in F1 Microbeads via 3D Confocal Microscopy**


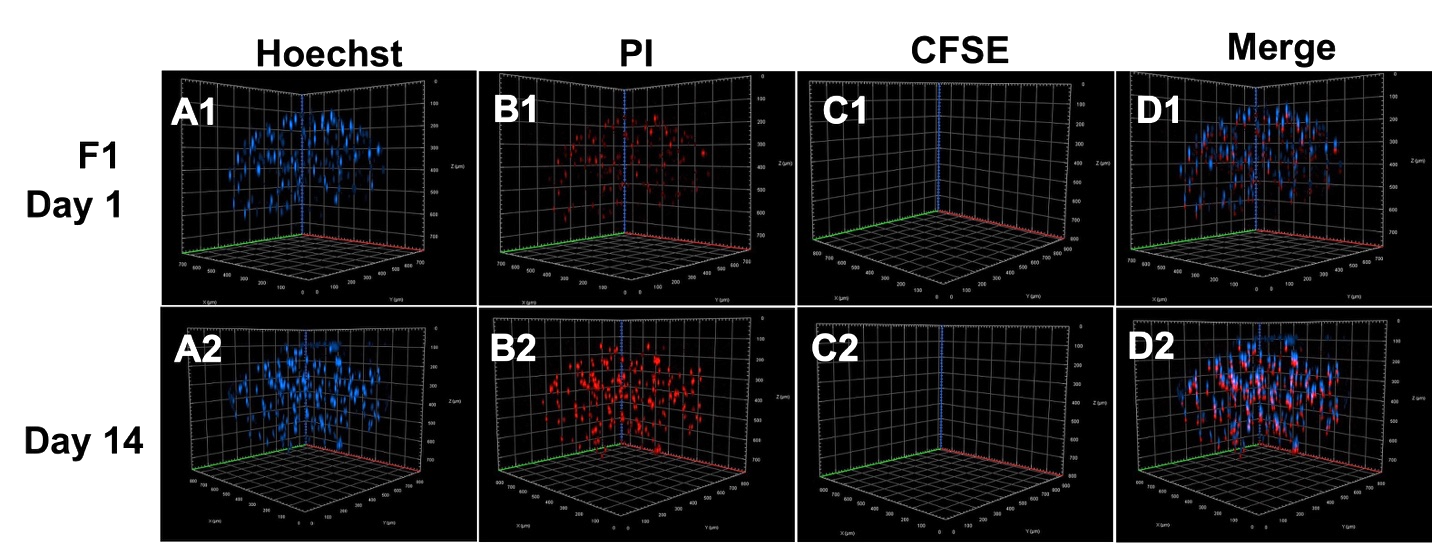


**Figure S4.** Representative 3D confocal microscopy images showing encapsulated cell distribution and viability within F1 microbead encapsulating imHCs and blank PLGA microspheres on day 1 (A1-D1) and day 14 (A2-D2). Hoechst (blue) marks nuclei, PI (red) labels dead cells, and CFSE (green) indicates live, metabolically active hMSCs. On day 1, the minimal PI signal suggests low initial cell death; however, CFSE fluorescence was absent, indicating the absence of encapsulated hMSCs. By day 14, the PI signal markedly increased.

**5. Assessment of Cell Viability and Distribution in F2 Microbeads via 3D Confocal Microscopy**


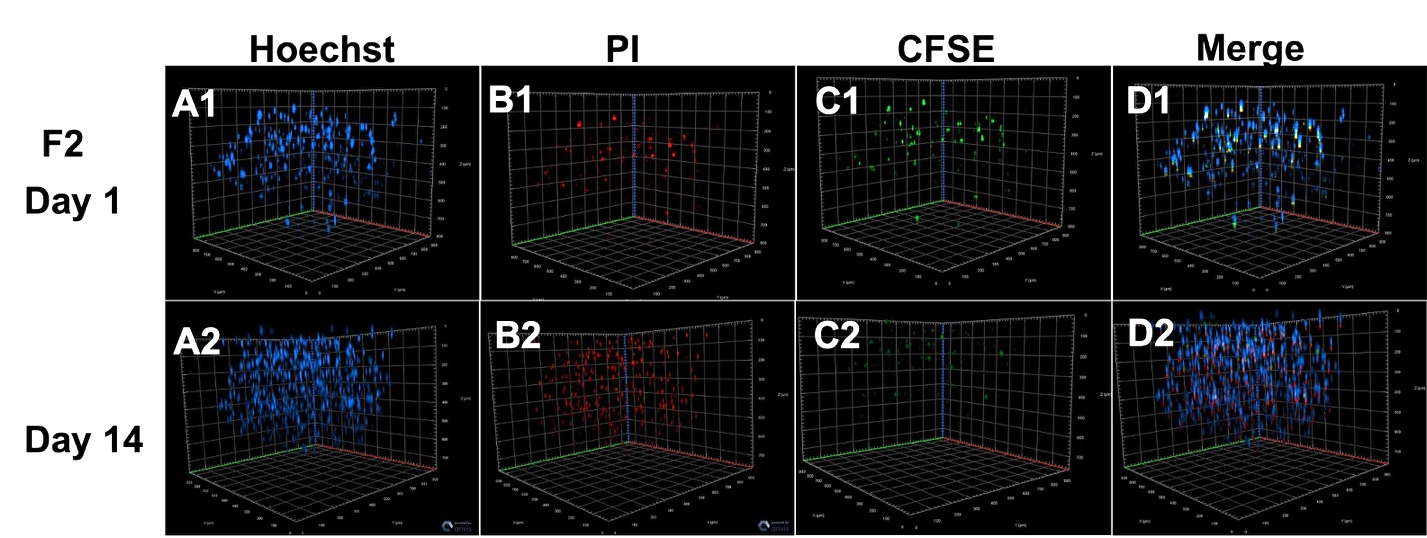


**Figure S5.** Representative 3D confocal microscopy images showing encapsulated cell distribution and viability within F2 microbeads encapsulating imHCs, hMSCs, and blank PLGA microspheres on day 1 (A1-D1) and day 14 (A2-D2). Hoechst (blue) stains all nuclei, propidium iodide (PI, red) marks dead cells, and CFSE (green) labels live, metabolically active hMSCs. On day 1, moderate green fluorescence and low PI signal indicate good initial cell viability. By day 14, increased red fluorescence and decreased CFSE signal reflect reduced metabolic activity and progressive cell death.

**6. Fluorescence Imaging of Cell Viability in Microbead Formulations**


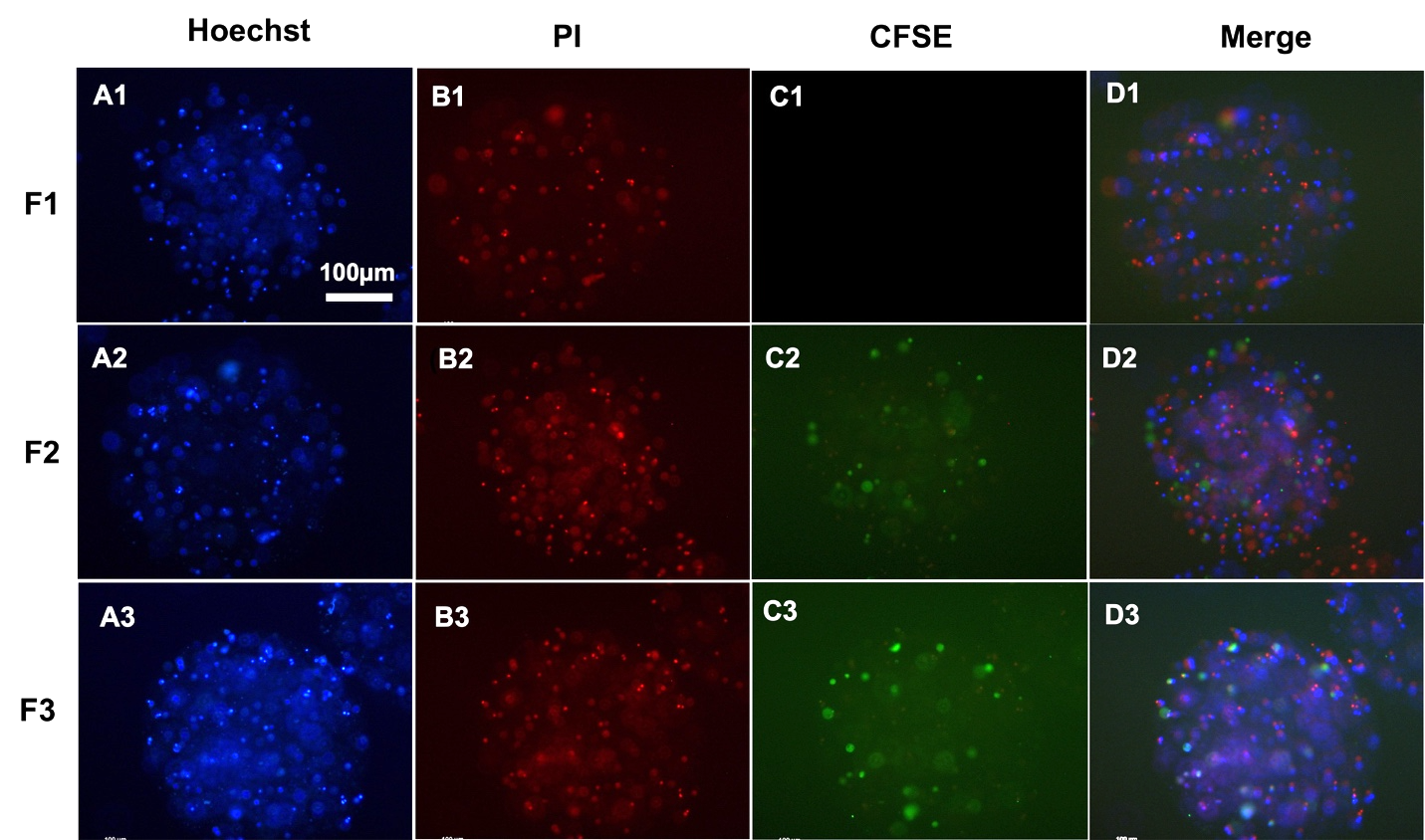


**Figure S6.** Representative fluorescence microscopy images of encapsulated cells within different microbead formulations (F1-F3) on day 14. Hoechst-stained nuclei (blue) identify all cells (A1-A3). PI staining (red) marks dead cells (B1-B3). CFSE staining (green) indicates live, metabolically active hMSCs (C1-C3). Merged images (D1-D3).


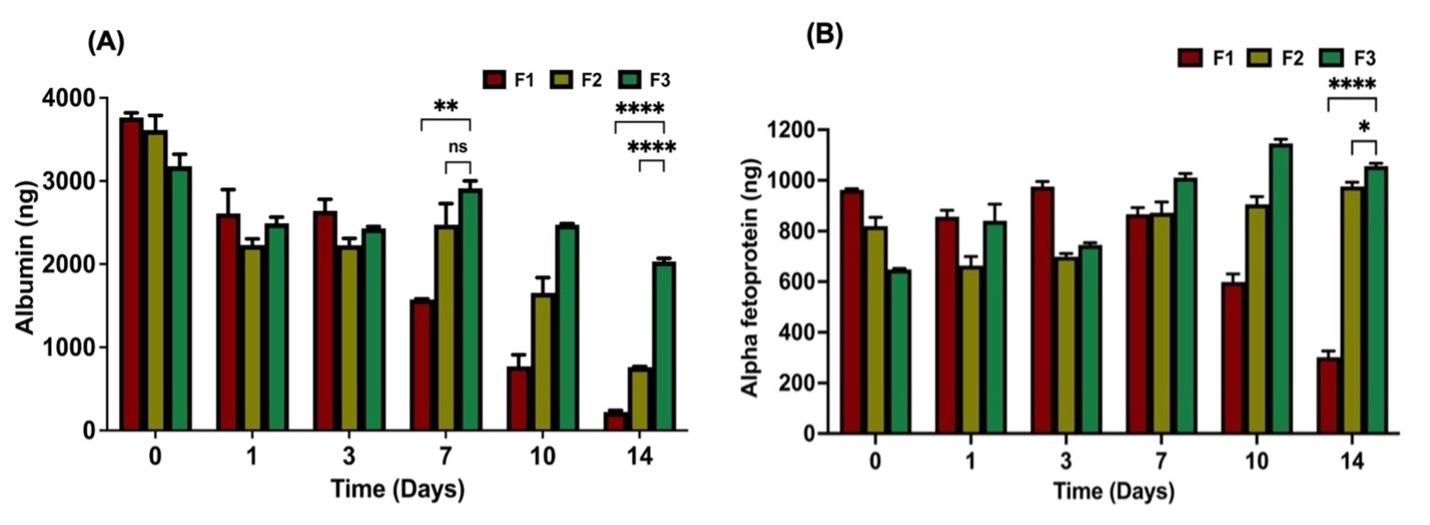
**7. Albumin and AFP Secretion from Encapsulated Hepatocytes**

**Figure S7.** Albumin and AFP secretion from encapsulated immortalized hepatocytes over 14 days in three microbead formulations (F1-F3). (A) Albumin secretion declined over time in all groups. F3 retained significantly higher levels on day 7 (2,910.53 ± 74.74 ng) compared to F2 (2,475.27 ± 206.31 ng, p = 0.05) and F1 (1,574.80 ± 6.37 ng, p < 0.01), and maintained superior secretion on day 14 (F3: 2,032.53 ± 29.45 ng; F2: 761.80 ± 6.21 ng; F1: 221.87 ± 14.01 ng; p < 0.0001). (B) AFP secretion increased over time, with F3 showing significantly higher levels by day 14 (1,057.00 ± 9.19 ng) compared to F2 (976.00 ± 3.90 ng, p < 0.05) and F1 (302.00 ± 20.06 ng, p < 0.0001).
